# Early Developmental EEG and Seizure Phenotypes in a Full Gene Deletion of Ubiquitin Protein Ligase E3A Rat Model of Angelman Syndrome

**DOI:** 10.1523/ENEURO.0345-20.2020

**Published:** 2021-03-23

**Authors:** Heather A. Born, Luis A. Martinez, Amber T. Levine, Sarah E. Harris, Shubhangi Mehra, Wai Ling Lee, Scott V. Dindot, Kevin R. Nash, Jill L. Silverman, David J. Segal, Edwin J. Weeber, Anne E. Anderson

**Affiliations:** 1 Cain Foundation Laboratories, Jan and Dan Duncan Neurological Research Institute at Texas Children’s Hospital, Houston, TX 77030; 2 Department of Pediatrics, Baylor College of Medicine, Houston, TX 77030; 3 Rice University, Houston, TX 77005; 4 Department of Veterinary Pathobiology, College of Veterinary Medicine and Biomedical Sciences, Texas A&M University, College Station, TX 77843; 5 Department of Molecular Pharmacology and Physiology, University of South Florida, Tampa, FL 33612; 6 MIND Institute and Department of Psychiatry and Behavioral Sciences, University of California Davis School of Medicine, Sacramento, CA 95817; 7 Departments of Neuroscience and Neurology, Baylor College of Medicine, Houston, TX 77030

**Keywords:** Angelman syndrome, epilepsy, epileptiform activity, quantitative EEG, seizures, spectral power

## Abstract

Angelman syndrome (AS) is a neurodevelopmental disorder with unique behavioral phenotypes, seizures, and distinctive electroencephalographic (EEG) patterns. Recent studies identified motor, social communication, and learning and memory deficits in a CRISPR engineered rat model with a complete maternal deletion of the *Ube3a* gene. It is unknown whether this model recapitulates other aspects of the clinical disorder. We report here the effect of *Ube3a* maternal deletion in the rat on epileptiform activity, seizure threshold, and quantitative EEG. Using video-synchronized EEG (vEEG) monitoring, we assessed spectral power and epileptiform activity early postnatally through adulthood. While EEG power was similar to wild-type (WT) at 1.5 weeks postnatally, at all other ages analyzed, our findings were similar to the AS phenotype in mice and humans with significantly increased δ power. Analysis of epileptiform activity in juvenile and adult rats showed increased time spent in epileptiform activity in AS compared with WT rats. We evaluated seizure threshold using pentylenetetrazol (PTZ), audiogenic stimulus, and hyperthermia to provoke febrile seizures (FSs). Behavioral seizure scoring following PTZ induction revealed no difference in seizure threshold in AS rats, however behavioral recovery from the PTZ-induced seizure was longer in the adult group with significantly increased hippocampal epileptiform activity during this phase. When exposed to hyperthermia, AS rat pups showed a significantly lower temperature threshold to first seizure than WT. Our findings highlight an age-dependence for the EEG and epileptiform phenotypes in a preclinical model of AS, and support the use of quantitative EEG and increased δ power as a potential biomarker of AS.

## Significance Statement

Angelman syndrome (AS) is a severe developmental disorder associated with developmental delays, lack of speech, motor and coordination problems, intellectual disability, epilepsy, and abnormal electroencephalographic (EEG) patterns. Here, we present novel findings that the complete deletion of ubiquitin protein ligase E3A (*Ube3a*), which is an established cause of AS, results in age-dependent increased δ power, epileptiform activity, and altered seizure threshold in the AS rat model. Use of the rat model facilitates early developmental studies, including quantitative EEG analysis and hyperthermia-induced seizures. Our findings support use of the rat model for future preclinical studies targeting the development of new, more effective treatment options as well as use of the AS EEG phenotype as a quantitative and translatable biomarker.

## Introduction

Angelman syndrome (AS) is a rare developmental disorder associated with severe epilepsy, distinctive electroencephalographic (EEG) patterns, developmental delay, cognitive impairment, and motor dysfunction. The most common molecular mechanism that causes AS is deletion of the 15q11-q13 chromosomal region, which includes the paternally imprinted *UBE3A* gene and several additional genes, such as *GABRB3*, *GABRA5*, and *GABRG3*, which encode GABA_A_ receptor subunits. *UBE3A* encodes ubiquitin protein ligase E3A, which labels proteins for degradation. Loss of function through deletion or mutation of the maternal allele for *UBE3A*, the copy that is actively expressed in neurons while the paternal allele is silent, reduces UBE3A protein expression in the brain (Pembrey et al., 1989; Smith et al., 1992). The intact paternal copy is silenced by the *UBE3A* antisense (*UBE3A-AS*) transcript (Rougeulle et al., 1998; Meng et al., 2012). Loss of *UBE3A* expression can also result from defects in imprinting or paternal uniparental disomy. However, the severity of symptoms, including epilepsy and qEEG phenotypes, can be variable and affected by other differences including mosaic imprinting (milder symptoms) or haploinsufficiency of other genes with larger 15q11-q13 region deletions that encompass *UBE3A* and the GABA_A_ receptor subunits (typically more severe phenotypes exacerbated by non-*UBE3A* pathophysiology; Minassian et al., 1998; Lossie et al., 2001; Frohlich et al., 2019a). The most common seizure types in AS are atypical absences, myoclonic, generalized tonic-clonic, and atonic seizures (Galvan-Manso et al., 2005; Khan et al., 2019). The initial presentation may be the onset of febrile seizures (FSs), and many of these individuals then go on to develop afebrile seizures at a later age (Galvan-Manso et al., 2005). The EEG in humans with AS is characterized by high-amplitude slow waves and a lack of normal awake background rhythms, with the majority of individuals showing epileptiform discharges including focal, multifocal or generalized sharp waves, or spike-and-wave potentials even when there is not an ongoing clinical seizure (Boyd et al., 1988; Galvan-Manso et al., 2005).

AS-like phenotypes are present in *Ube3a* maternally deficient mice, including reduced seizure threshold, aberrant EEG activity (intermittent rhythmic δ and θ slow waves, epileptiform discharges), hypoactivity, and motor learning and coordination deficits (Jiang et al., 1998a; Huang et al., 2013; Judson et al., 2016; Sidorov et al., 2017). Currently, no cure is available and improved disease-modifying treatment options are critical, particularly with respect to abnormal EEG activity and seizures, which are frequently associated with AS. Epilepsy is present in ∼80% of the AS population. The seizures in AS are often drug-resistant, negatively impact quality of life, and can further disrupt sleep and cognition (Laan et al., 1998; Minassian et al., 1998; Vendrame et al., 2012), and recent work has identified quantitative EEG abnormalities in humans with AS compared with age-matched neurotypical groups (Sidorov et al., 2017; Frohlich et al., 2019a).

Translating preclinical studies to therapeutics has been made more difficult by the limitations of mouse models, as *Ube3a* maternally deficient mice show strain-dependent, inconsistent, and varying seizure and behavioral phenotypes (Jiang et al., 1998b; Huang et al., 2013; Mandel-Brehm et al., 2015; Born et al., 2017). Thus, highlighting a need for preclinical models that reliably mimic features of AS. In contrast with the many AS mouse models on different background strains, the only currently available rat AS model is on an outbred Sprague Dawley background. As the AS rat model has only been examined on this background at this point, it remains to be determined what the impact of different rat background strains would be on phenotypic expression. Recent advances in targeted nuclease technology has facilitated the development of genetic rat models, which may provide a number of benefits such as better predicting drug efficacy with more clinically relevant pharmacokinetics, and may more closely model sophisticated, complex behaviors in the domain of social interaction and cognition (Ellenbroek and Youn, 2016). The use of rats enables the testing of early developmental and neurophysiological studies.

To generate an improved model for understanding the underlying mechanisms of disease and translational studies of novel therapeutics, a novel AS rat model was developed using CRISPR technology that has a 90-kb deletion of the complete *Ube3a* gene (Berg et al., 2020; Dodge et al., 2020). Recent studies identified AS-relevant behavioral and neuroanatomical phenotypes in the new model, including delayed development of reflexes, decreased brain volume, deficits in motor ability, abnormal social behavior and communication, and decreased performance on learning and memory tasks (Berg et al., 2020; Dodge et al., 2020). Building on these prior studies using the novel rat AS model, we evaluated EEG activity and seizure threshold beginning early postnatally and into adulthood in AS compared with wild-type (WT) rats. We assessed δ power from cortical and hippocampal recordings during both light and dark cycles as well as frequency of epileptiform activity. We also took advantage of the rat model to evaluate vulnerability to multiple mechanisms for evoking seizures, including the chemoconvulsant pentylenetetrazol (PTZ) and audiogenic stimuli, similar to what has been previously evaluated in AS mouse models. We extended these studies by also evaluating a more clinically-relevant method for evoked seizures through early postnatal exposure to hyperthermia, which is an established model for FSs.

## Materials and Methods

### Animals

We maintained colonies of *Ube3a* maternal deficient (*Ube3a^m−/p+^*) rats and WT (*Ube3a^m+/p+^*) rats on a Sprague Dawley background for EEG and seizure threshold experiments in the rat vivarium. Recent publications describe in more depth the generation of the novel AS rat (Berg et al., 2020; Dodge et al., 2020). Briefly, the AS rat model has a complete maternal deletion of all exons of the *Ube3a* gene and generates a null mutation. Our colony has been maintained using paternal deletion rats (*Ube3a^m+/p-^*) generated by crossing male paternal deletion (*Ube3a^m+/p-^*) rats with WT female rats. Maternal deletion experimental rats and WT littermates were generated using female paternal deletion (*Ube3a^m+/p-^*) rats paired with WT male rats. In all experiments, we used sex-balanced pup, juvenile, and adult *Ube3a^m−/p+^* and WT littermates at the ages described. Rats were identified and tissue collected for genotyping using ear hole markings before weaning. Genotyping was performed with GoTaq Green Master Mix (Promega Corporation) using the following primers: Rube1123, TAGTGCTGAGGCACTGGTTCAGAGC; Rube1606r, TGCAAGGGGTAGCTTACTCATAGC; Ub3aDelSpcfcF6, ACCTAGCCCAAAGCCATCTC, and Ub3aDelR2, GGGAACAGCAAAAGACATGG (Integrated DNA Technologies). Rats used for seizure induction studies were group housed and tested during the light cycle. Before weaning, rats recorded for EEG activity were kept with the nursing dam in between recording sessions. Adult rats recorded for EEG activity were singly housed. Rats were provided with food *ad libitum* on corncob bedding and housed in rooms with a 14/10 h light/dark cycle at 22°C. Animal care and use were in accordance with regulations from the National Institutes of Health *Guidelines for the Care and Use of Laboratory Animals* and approved by the Institutional Animal Care and Use Committee at Baylor College of Medicine.

### Electrode implantation

WT and AS rats were implanted for EEG recordings using two cortical screw electrodes and one hippocampal depth electrode (Plastics One). For early age studies, rats were implanted at postnatal day (P)8–P9. After anesthesia with isoflurane and positioning the rats in a stereotaxic frame, a small midline sagittal incision was made on the scalp. The cortical recording electrodes (stereotaxic coordinates relative to bregma: bilateral placement at −0.5 mm posterior, ±2.2 mm lateral; Baram et al., 1997) and reference electrode anterior to the bregma were implanted subdurally through small holes drilled in the skull. The hippocampal electrode was positioned −2.2 mm posterior, +2.5 mm lateral, at the depth of −2.5 mm. These electrodes were held in place with Metabond (Parkell) and dental cement (Co-Oral-Ite Dental Mfg). The ground electrode was sutured in the cervical paraspinous area. All electrodes were inserted into a six-channel pedestal and connected to the commutator for recording. Rats were provided with slow-release buprenorphine and lidocaine/bupivacaine for pain relief. Animals implanted after weaning were implanted at three to four weeks of age using the above process with two subdural electrodes over somatosensory cortex positioned at coordinates (determined relative to bregma: bilateral placement at −1.0 mm posterior, ±3.0 mm lateral) and the hippocampal-depth electrode positioned in area CA1 (−4.0 mm posterior, 2.8 mm lateral, at the depth of −2.8 mm). Postweaning animals are provided additional pain management with Rimadyl tablets for 24 h presurgery, day of surgery, and 24 h postsurgery and allowed to recover from surgery and the effects of analgesia for at least one week before long-term recording.

### EEG acquisition

Video-synchronized EEG (vEEG) was recorded and analyzed using the Nicolet system (Natus) and Labchart V8 software (AD Instruments). EEG data were acquired at a sampling rate of 2000 Hz, filtered (bandpass: 0.5–70 Hz; notch), and reviewed blind to genotype for spectral power and epileptiform activity analysis. Before weaning at three weeks, pup and juvenile rats recorded for EEG activity were monitored for 1.5-h blocks (30-min acclimatization, 1-h recording) and then returned to dam for nursing. For postweaning recordings, after 30-min acclimatization to the recording chamber, rats were recorded for 24 h/d for at least 48 h per session.

### Spectral power analysis

In order to quantify differences between WT and AS rats during normal, baseline EEG activity, we used a fast Fourier transform (FFT) algorithm to convert electrical activity to frequency with a FFT size of 2042 using the Hann (cosine-bell) method and a 50% window overlap (Cooley and Tukey, 1965). After visually inspecting the vEEG recordings and manually excluding artifacts from the data, we used Labchart V8 software for spectral analysis of power in WT and AS rats age-matched for time of electrode implantation (1.5 weeks: *n* = 9/group; two weeks: *n* = 7–10/group; three weeks: *n* = 7–12/group; 4.5 months: *n* = 4–6/group). In addition to comparing across individual frequencies, we also quantified the power for δ (0.5–4 Hz), θ (5–8 Hz), α (8–12 Hz), β (14–29 Hz), and γ (30–50 Hz) frequency bands (Table 1). For the 1.5- and two-weeks age groups, activity was recorded during the afternoon in the light cycle. For the three- and 4.5-month age groups, analysis of power was performed using an unbiased time of day approach for 2 h of recording between 12 and 2 P.M. as a representation of activity during the light cycle and 12 and 2 A.M. as a representation of activity during the dark cycle.

**Table 1:** Summary of EEG power quantified by wave band

Figure	Genotype	δ (μV^2^)	θ (μV^2^)	α (μV^2^)	β (μV^2^)	γ (μV^2^)
1*B*	WT	297.76 ± 40.50	63.38 ± 10.52	46.18 ± 6.47	125.78 ± 27.60	18.08 ± 5.36
	AS	282.57 ± 57.07	49.22 ± 10.82	35.49 ± 7.37	90.87 ± 27.47	14.19 ± 5.80
1*D*	WT	506.92 ± 111.63	216.80 ± 47.61	112.64 ± 25.44	156.27 ± 38.40	104.94 ± 24.61
	AS	727.85 ± 104.84	301.46 ± 32.85	144.31 ± 12.74	180.67 ± 13.08	107.91 ± 10.30
1*F*	WT	744.89 ± 140.51	216.41 ± 36.68	100.26 ± 18.82	115.86 ± 25.90	59.42 ± 14.10
	AS	1281.13 ± 296.51	421.51 ± 130.40	191.36 ± 74.02	201.43 ± 79.00	84.49 ± 37.57
1*H*	WT	688.74 ± 102.11	331.59 ± 36.73	212.46 ± 19.94	203.75 ± 17.47	62.82 ± 8.05
	AS	900.24 ± 150.71	414.01 ± 77.45	254.16 ± 48.22	245.98 ± 43.30	77.94 ± 13.85
1-2*B*	WT	540.49 ± 140.59	229.37 ± 54.89	111.16 ± 30.06	125.89 ± 38.02	59.99 ± 18.67
	AS	767.54 ± 140.60	269.44 ± 44.37	120.26 ± 20.44	126.26 ± 22.82	48.22 ± 8.37
1-2*D*	WT	517.14 ± 166.06	231.12 ± 77.33	127.11 ± 39.81	143.99 ± 22.56	82.58 ± 11.88
	AS	564.18 ± 105.97	190.36 ± 30.54	101.79 ± 14.86	135.78 ± 20.36	99.40 ± 16.73
2*B*	WT	2373.34 ± 521.73	318.86 ± 90.85	131.22 ± 30.45	235.07 ± 62.74	25.02 ± 7.88
	AS	2808.20 ± 863.86	295.10 ± 89.59	120.04 ± 32.49	195.10 ± 67.63	29.43 ± 9.56
2*D*	WT	5047.64 ± 949.37	1514.25 ± 286.06	506.09 ± 89.07	497.79 ± 81.19	205.82 ± 39.83
	AS	7731.04 ± 1263.57	2367.36 ± 421.52	729.52 ± 137.89	622.39 ± 113.31	208.68 ± 30.75
2*F*	WT	10,253.67 ± 1807.28	3459.56 ± 485.86	1209.27 ± 150.89	1004.03 ± 135.55	338.55 ± 53.40
	AS	16,437.81 ± 2925.61	4898.81 ± 551.14	1562.46 ± 148.64	1179.09 ± 149.27	354.03 ± 65.44
2*H*	WT	7728.26 ± 3723.85	3924.04 ± 1169.57	2358.63 ± 662.04	1648.54 ± 248.16	396.74 ± 120.99
	AS	12,888.01 ± 2879.10	5739.52 ± 731.21	3309.58 ± 323.23	2438.88 ± 344.48	496.97 ± 64.32
2-1*B*	WT	7508.69 ± 2192.19	2648.74 ± 537.71	886.79 ± 136.76	886.79 ± 138.71	311.73 ± 63.08
	AS	12,199.45 ± 1458.45	4173.65 ± 436.20	1364.89 ± 126.85	1047.68 ± 117.14	310.29 ± 38.31
2-1*D*	WT	3600.24 ± 106.84	22,28.29 ± 257.55	1116.51 ± 256.97	1163.53 ± 181.13	614.78 ± 232.00
	AS	4851.45 ± 747.34	2497.32 ± 320.49	1419.37 ± 188.39	1337.88 ± 215.74	538.22 ± 58.13

EEG power quantified for δ (0.5–4 Hz), θ (5–8 Hz), α (8–12 Hz), β (14–29 Hz), and γ (30–50 Hz) frequency bands.

### Epileptiform activity analysis

Data from the recordings used for spectral power analysis were manually evaluated and quantified for the percent time spent in epileptiform activity at the juvenile (three weeks: *n* = 7–11/group) and adult (4.5 months: *n* = 6–9/group) age groups. In our recordings, we observed spikes, which were defined as waveforms <200 ms in duration and with a peak amplitude ≥2× baseline background amplitude, and polyspikes, defined as spikes crossing the baseline more than two times. Clinical epileptiform activity also includes rhythmic 3-Hz spike-and-wave, slow spike-and-wave, atypical paroxysmal fast activity, and hypsarrhythmic patterns, however these patterns were not evident in the recordings from the AS rats. To quantify percent time spent in epileptiform activity, we selected 2-min epochs every 10 min from 12 to 1 A.M. (dark cycle) and 12 to 1 P.M. (light cycle) to evaluate representative activity during continuous vEEG monitoring and counted the amount of time (in seconds) that included epileptiform activity. Wakeful, non-moving periods of recording were used for analysis to avoid contamination with artifact from electromyographic (EMG) activity, movement, or grooming behaviors, which were determined using simultaneous video and EMG recordings. Data were reported as percentages of total observation time. vEEG recordings were also reviewed for absence-like (electrographic seizures accompanied by behavioral arrest) and motor seizures (electrographic seizures accompanied by motor changes involving myoclonic jerks, tonic-clonic activity, wild running, and loss of postural control).

### Audiogenic seizure (AGS) induction

We tested the AGS susceptibility of WT and AS rats at multiple ages during development (two weeks: *n* = 6–10/group; one month: *n* = 12/group; 4.5 months: *n* = 14/group) using a 130-dB emitting personal/door alarm. For the tests, individual rats were placed in a clean, empty cage in a sound-attenuating chamber and habituated for 5 min, observed for 1 min with no sound played, then observed for behavioral changes while the alarm was sounded for 2 min and after the cessation of the alarm. Using video recorded during testing, a blinded observer recorded the appearance of and latency to the onset of immobility (severely attenuated behavior) and any wild running or tonic-clonic behavior. The latency to recovery to first movement following the end of the alarm sound was determined by evaluating the time to first complete paw lifted from the cage surface or grooming behavior (Mandel-Brehm et al., 2015).

### PTZ induction

For comparison of seizure threshold in the WT and AS rats, we used a dose of 50 mg/kg intraperitoneal PTZ (Sigma-Aldrich) that had been evaluated for previous studies from the lab and determined to typically induce a single generalized, motor seizure in the majority of WT Sprague Dawley rats tested at a juvenile age with a high survival rate. For each age group (1.5 weeks: *n* = 7–16/group; one month: *n* = 17/group; 4.5 months: *n* = 14/group), rats were first habituated to a clean, empty cage for 30 min, injected with PTZ, and monitored blind to genotype for a 1-h observation period. In-depth analysis of the latency to and duration of seizure behavior was scored using video recordings for the observation period. Seizure stage was determined based on a modified Racine scale (Lüttjohann et al., 2009), stage 1: rigid posture or immobility, mouth moving; stage 2: tail clonus; stage 3: partial body clonus with forelimb or hindlimb clonus, head bobbing; stage 4: rearing with whole body clonus while retaining posture; stage 5: rearing and falling; and stage 6: loss of posture, wild running, or jumping. The latency to first clonus measures the time to the first instance of any tail, limb, or head clonus (stage 2 or higher). The latency to first seizure measures the time to the onset of a generalized motor seizure (stage 4 or higher), while the latency to recovery was determined as the time until the first instance of a complete paw lifted from the cage surface or grooming following the end of generalized motor seizure behavior. A separate cohort of WT and AS animals (*n* = 3–5/group) first underwent surgery for electrode implantation, recovery, baseline EEG activity recording, and then seizure induction with PTZ (50 mg/kg, i.p.). One animal died as a result of PTZ induction and was not included in post-PTZ analysis. The percent time spent in epileptiform activity was evaluated at 30 min after PTZ induction using a 5-min epoch and at 3 h after PTZ induction using a 10-min epoch. Animals continued to be monitored for at least 1 d following seizure for epileptiform activity.

### Hyperthermic-evoked seizure induction

FS were induced in WT and AS rats at the age of P10–P 11 by gradually raising internal temperature 2°C/min using a heat lamp (TCAT-2LV; Physitemp Instruments LLC.) until behavioral seizures characterized by immobility and mouth and limb clonus were observed or ∼41.5°C rectal temperature was reached (*n* = 5–10/group) and then recovered to baseline temperature (32–33°C) before return to dam (Baram et al., 1997; Dubé et al., 2009). The severity of seizure behavior was scored using the modified Racine scale (Lüttjohann et al., 2009), and the duration of time spent in single or bilateral forelimb clonus (the most commonly observed behaviors) was observed. To verify that behavioral seizure activity was associated with electrographic seizures, an additional cohort of WT and AS animals were implanted for EEG activity at P8–P9, returned to nursing dams to recover, and exposed to hyperthermia or normothermia (room temperature air) at P10–P11 and observed for EEG and behavioral activity. At P10–P11, baseline EEG activity was recorded for 1.5 h, pups returned to dam for nursing, then recorded for a second block as follows: 30-min acclimatization, hyperthermia or normothermia induction, and ∼30 min postinduction.

### Western blotting

Western blotting on whole hippocampal tissue was performed using methods described previously (*n* = 5/group; Brewster et al., 2013). Homogenates were loaded into SDS-PAGE gels, separated by electrophoresis, transferred to Hybond-P polyvinyl difluoride membranes (GE Healthcare), blocked, and probed for antibodies (Nguyen et al., 2015; Carter et al., 2017). Optical densities of all immunoreactive bands were visualized with ECL Plus and expression levels quantified using Image Studio Lite, with Ube3a levels normalized to GAPDH levels as a loading control. Results were expressed as percentages of age-matched WT rats. The primary antibodies used for western blotting were as follows: Ube3a/E6-AP at 1:1000 (Sigma E8655) and GAPDH at 1:10,000 (EMD Millipore).

### Statistics

All statistical analyses were done using GraphPad Prism 8.0. Student’s *t* test was used to compare between genotypes for percent time in epileptiform activity and seizure induction comparisons with Welch’s correction where variances were significantly different. EEG spectral analysis results were analyzed with two-way ANOVA and Sidak’s multiple comparisons for *post hoc* tests to determine differences between component frequencies. Group sizes were determined based on power analyses and previous studies from the lab. Data were assessed for outliers by group using the Grubb’s test. A table further detailing the statistical analyses used in our studies has been included (Table 2). For the spectral power data, graphs display group mean with errors bars ±SEM; *p < 0.05, **p < 0.01, ***p < 0.001, ****p < 0.0001. For two group comparisons, the raw data were input on https://www.estimationstats.com/, which generated Gardner–Altman estimation plots for two-independent-group mean differences (Ho et al., 2019). For each graph, the raw data are plotted on the left axis with the means plotted as solid lines and the mean difference plotted on the right axis as a dot with 95% confidence intervals indicated by the ends of the vertical error bar. The mean difference is plotted as a bootstrap sampling distribution with 5000 bootstrap samples taken and the confidence interval bias-corrected and accelerated.

**Table 2.** Summary of statistical tests

				*Post hoc* comparisons				
Figure	Test	Results	*p* value		95.00% CI of diff.	Mean 1	Mean 2	Sig.
1*B*	Two-way ANOVA	Interaction: *F*_(50,816)_ = 0.1519	>0.9999					
		Frequency: *F*_(50,816)_ = 31.82	<0.0001					
		Genotype: *F*_(1,816)_ = 2.519	0.1129					
1*D*	Two-way ANOVA	Interaction: *F*_(50,765)_ = 1.637	0.0043	1 Hz	−85.94 to −8.531	134.17	181.41	**
		Frequency: *F*_(50,765)_ = 47.27	<0.0001	2 Hz	−99.88 to −22.47	146.01	207.19	****
		Genotype: *F*_(1,765)_ = 18.97	<0.0001	3 Hz	−95.39 to −17.98	113.21	169.89	****
				4 Hz	−85.79 to −8.374	87.19	134.27	**
1*F*	Two-way ANOVA	Interaction: *F*_(50,561)_ = 2.252	<0.0001	1 Hz	−227.3 to −66.59	214.79	361.74	****
		Frequency: *F*_(50,561)_ = 30.41	<0.0001	2 Hz	−245.6 to −84.88	220.65	385.89	****
		Genotype: *F*_(1,561)_ = 29.53	<0.0001	3 Hz	−199.9 to −39.24	157.28	276.87	****
				4 Hz	−162.9 to −2.170	109.55	192.08	*
1*H*	Two-way ANOVA	Interaction: *F*_(50,408)_ = 1.149	0.2358	1 Hz	−129.1 to −21.47	191.40	266.67	***
		Frequency: *F*_(50,408)_ = 41.52	<0.0001	2 Hz	−128.2 to −20.64	184.40	258.83	***
		Genotype: *F*_(1,408)_ = 13.53	0.0003					
1-1*B*	Unpaired *t* test with Welch’s correction	*t* = 10.40, df = 4.063; 95% CI: −1.208 to −0.7016	0.0004					
1-2*B*	Two-way ANOVA	Interaction: *F*_(50,867)_ = 0.9298	0.6134	1 Hz	−114.5 to −9.244	142.99	204.87	**
		Frequency: *F*_(50,867)_ = 29.69	<0.0001	2 Hz	−124.7 to −19.43	160.32	232.38	***
		Genotype: *F*_(1,867)_ = 5.405	0.0203					
1-2*D*	Two-way ANOVA	Interaction: *F*_(50,408)_ = 0.7116	0.9303	1 Hz	−134.8 to −34.49	114.52	199.17	****
		Frequency: *F*_(50,408)_ = 18.65	<0.0001					
		Genotype: *F*_(1,408)_ = 0.7579	0.3845					
2*B*	Two-way ANOVA	Interaction: *F*_(50,765)_ = 0.2408	>0.9999					
		Frequency: *F*_(50,765)_ = 20.50	<0.0001					
		Genotype: *F*_(1,765)_ = 0.4037	0.5254					
2*D*	Two-way ANOVA	Interaction: *F*_(50,765)_ = 1.665	0.0033	1 Hz	−1193 to −269.8	1747.29	2478.75	****
		Frequency: *F*_(50,765)_ = 38.40	<0.0001	2 Hz	−1232 to −308.9	1407.57	2178.16	****
		Genotype: *F*_(1,765)_ = 15.16	0.0001	3 Hz	−1050 to −126.7	876.49	1464.83	**
2*F*	Two-way ANOVA	Interaction: *F*_(50,561)_ = 2.855	<0.0001	1 Hz	−2614 to −986.7	2801.60	4602.08	****
		Frequency: *F*_(50,561)_ = 52.60	<0.0001	2 Hz	−2674 to −1047	3021.36	4881.72	****
		Genotype: *F*_(1,561)_ = 21.59	<0.0001	3 Hz	−2135 to −507.5	2254.47	3575.70	****
				4 Hz	−1687 to −59.74	1626.22	2499.73	*
2*H*	Two-way ANOVA	Interaction: *F*_(50,255)_ = 0.8056	0.8194	1 Hz	−2861 to −279.7	2253.00	3823.60	**
		Frequency: *F*_(50,255)_ = 13.37	<0.0001	2 Hz	−2768 to −186.7	2214.00	3691.60	**
		Genotype: *F*_(1,255)_ = 10.13	0.0016					
								
2-1*B*	Two-way ANOVA	Interaction: *F*_(50,867)_ = 2.797	<0.0001		−1659 to −404.5	2472.56	3504.52	****
		Frequency: *F*_(50,867)_ = 49.22	<0.0001		−2014 to −759.1	2199.18	3585.74	****
		Genotype: *F*_(1,867)_ = 26.10	<0.0001	3 Hz	−1828 to −572.9	1373.97	2574.32	****
				4 Hz	−1512 to −257.5	965.80	1850.69	***
				5 Hz	−1265 to −9.782	825.34	1462.54	*
2-1*D*	Two-way ANOVA	Interaction: *F*_(50,306)_ = 1.048	0.3937	1 Hz	−733.2 to −164.3	1002.67	1451.40	****
		Frequency: *F*_(50,306)_ = 46.53	<0.0001	2 Hz	−605.8 to −36.91	1032.67	1354.00	*
		Genotype: *F*_(1,306)_ = 9.863	0.0019					
3*B*, dark cycle	Unpaired *t* test with Welch’s correction	*t* = 2.612, df = 10.98; 95% CI: 0.1150–1.347	0.0242					
3*B*, light cycle		*t* = 2.480, df = 11.70; 95% CI: 0.08133–1.287	0.0294					
3*D*, dark cycle	Unpaired *t* test with Welch’s correction	*t* = 2.296, df = 8.633; 95% CI: 0.01022–2.409	0.0485					
3*D*, light cycle	Unpaired *t* test with Welch’s correction	*t* = 2.116, df = 8.641; 95% CI: −0.1092–2.980	0.0647					
4*A*, latency clonus	Unpaired *t* test	*t* = 0.08745, df = 21; 95% CI: −138.9–127.7	0.9311					
4*A*, latency seizure	Unpaired *t* test with Welch’s correction	*t* = 0.4379, df = 9.041; 95% CI: −481.5–325.2	0.6717					
4*A*, recovery	Unpaired *t* test	*t* = 0.6691, df = 21; 95% CI: −1127–2197	0.5107					
4*B*, latency clonus	Unpaired *t* test	*t* = 0.4538, df = 28. 95% CI: −47.05–29.98	0.6534					
4*B*, latency seizure	Unpaired *t* test	*t* = 0.5004, df = 25; 95% CI: −13.37–21.95	0.6211					
4*B*, recovery	Unpaired *t* test	*t* = 1.693, df = 30; 95% CI: −18.96–202.6	0.1009					
4*C*, latency clonus	Unpaired *t* test	*t* = 1.605, df = 25; 95% CI: −18.48–2.295	0.1211					
4*C*, latency seizure	Unpaired *t* test	*t* = 0.7429, df = 23; 95% CI: −13.22–6.234	0.4651					
4*C*, recovery	Unpaired *t* test	*t* = 2.523, df = 19; 95% CI: 237.9–2552	0.0207					
4-1*A*, immobility	Unpaired *t* test	*t* = 0.1999, df = 14; 95% CI: −39.49–32.76	0.8444					
4-1*A*, recovery	Unpaired *t* test	*t* = 1.531, df = 14; 95% CI: −47.37–283.8	0.148					
4-1*B*, immobility	Unpaired *t* test	*t* = 2.074, df = 22; 95% CI: −69.50 to −0.002622	0.05					
4-1*B*, recovery	Unpaired *t* test	*t* = 3.054, df = 22; 95% CI: 113.7–595.3	0.0058					
4-1*C*, immobility	Unpaired *t* test	*t* = 0.5781, df = 26; 95% CI: −22.82–40.68	0.5682				
4-1*C*, recovery	Unpaired *t* test	*t* = 0.2767, df = 26; 95% CI: −57.80–44.09	0.7842					
5*C*, 30 min: cortical	Unpaired *t* test	*t* = 0.1210, df = 5; 95% CI: *t* = 0.1210, df = 5	0.9084					
5*C*, 30 min: hippocampal	Unpaired *t* test	*t* = 0.07701, df = 5; 95% CI: −22.03–20.75	0.9416					
5*C*, 3 h: cortical	Unpaired *t* test	*t* = 3.039, df = 5; 95% CI: 4.279–51.22	0.0288					
5*C*, 3 h hippocampal	Unpaired *t* test	*t* = 3.098, df = 5; 4.972–53.41	0.0269					
6*C*	Unpaired *t* test	*t* = 2.806, df = 13; 95% CI: −5.558 to −0.7225	0.0149					

*p < 0.05, **p < 0.01, ***p < 0.001, ****p < 0.0001.

### Data availability

The datasets generated and analyzed during the current study are available from the corresponding author on reasonable request.

## Results

### Increased δ power develops early in life in AS rats and persists into adulthood

Recently published work has shown loss of UBE3A protein expression in multiple brain regions as a result of the maternal *Ube3a* deletion (Dodge et al., 2020), which we confirmed in our colony using western blots with hippocampal tissue of adult AS rats (*p* < 0.0001; Extended Data Fig. 1-1). This model recapitulates many of the phenotypes present in human AS including developmental delays in reflexes, motor deficits, decreased social communication, and impaired learning and memory (Berg et al., 2020; Dodge et al., 2020). However, it was unknown whether the AS rat model also mirrored abnormal EEG patterns and increased epileptiform activity, both of which are frequently found in humans with AS.

**Figure 1.**
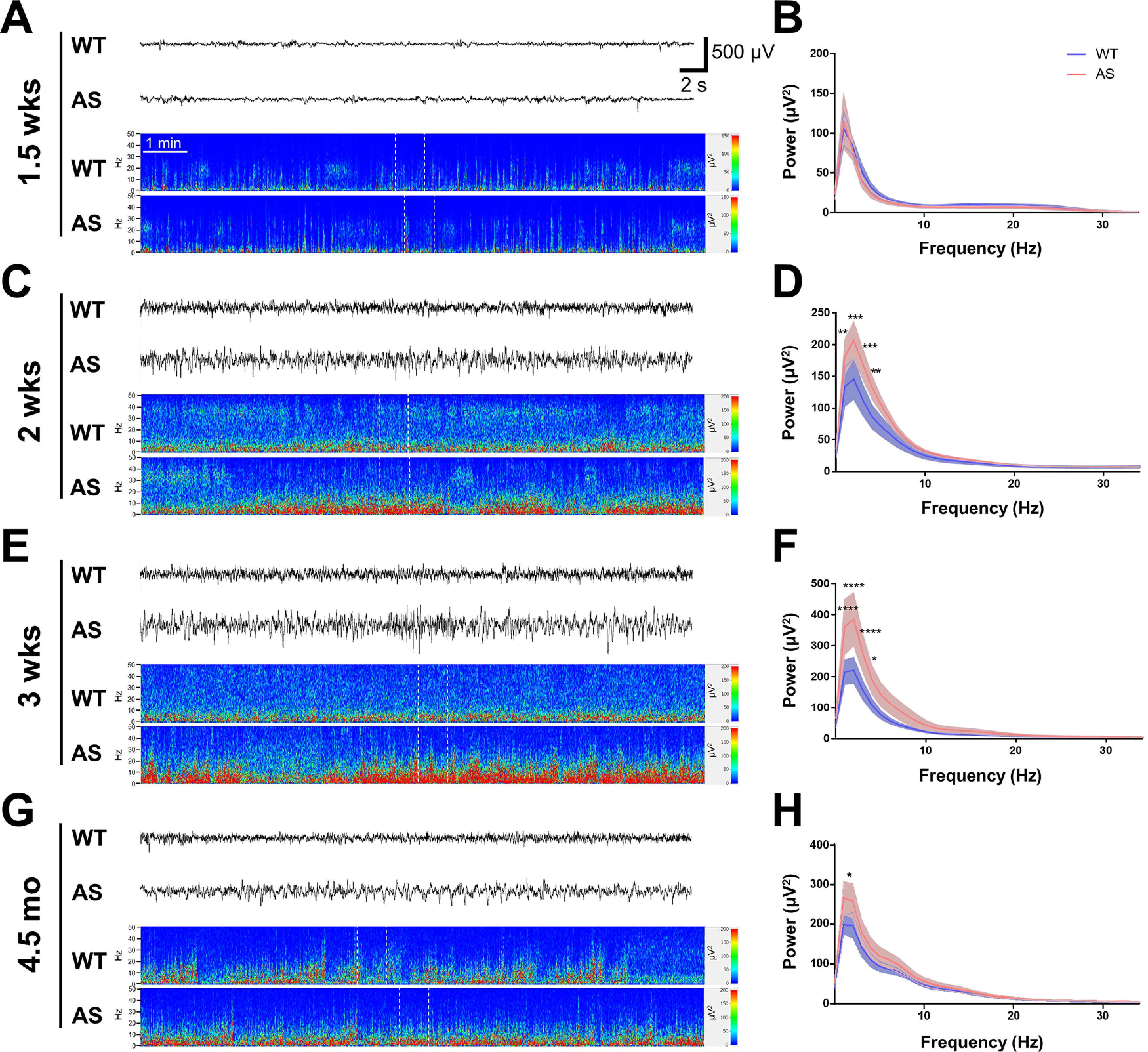
Characterization of cortical EEG activity from early development into adulthood recorded during the light cycle. ***A***, ***C***, ***E***, ***G***, Cortical EEG traces and power spectra show representative activity from neonates, juveniles, and adult WT and AS rats (with the corresponding EEG activity segment indicated by white dotted lines in each spectrogram), while ***B***, ***D***, ***F***, ***H*** show the age-matched quantification of EEG activity using spectral power analysis. ***A***, ***B***, Representative EEG activity and spectral power analysis from 1.5-week-old WT and AS rats show discontinuous activity typical of neonatal EEG recordings in both groups and spectral analysis of the epochs of continuous activity showed no significant difference between WT and AS rats (*n* = 9). ***C***, ***D***, Representative EEG activity and spectral power analysis from WT and AS rats at two weeks reveals an increase in δ power in AS compared with WT rats (*n* = 7–10; *p* < 0.01 at 1 Hz, *p* < 0.001 at 2–3 Hz, *p* < 0.01 at 4 Hz). ***E***, ***F***, Representative EEG activity and spectral power analysis at three weeks of age highlight an increase in δ power in AS rats compared with WT (*n* = 7–12; *p* < 0.001 at 1–3 Hz, *p* < 0.05 at 4 Hz). ***G***, ***H***, Representative EEG activity and spectral power at 4.5 months of age show the persistence of increased δ power in adult AS compared with WT rats (*n* = 4–6; *p* < 0.05 at 1 Hz); *p < 0.05, **p < 0.01, ***p < 0.001, ****p < 0.0001.

10.1523/ENEURO.0345-20.2020.f1-1 Extended Data Figure 1-1 Confirmation of Ube3a protein expression loss in the *Ube3a* maternal deficiency AS rat model. ***A***, Western blotting for Ube3a (Sigma E8655; 1:1000) with GAPDH used as a loading control confirms loss of expression in adult AS rat hippocampus. ***B***, The expression level of Ube3a is significantly decreased in hippocampal tissue from adult AS rats compared to age-matched WT littermates (*n* = 5, *p* < 0.0001). Download Figure 1-1, TIF file.

We performed comparisons of cortical EEG activity in AS rats compared with age-matched WT rats with the earliest time point at 1.5 weeks of age in rat pups ranging to 4.5 months of age in adult rats. Overall, qualitative examination of cortical EEG activity suggests AS rats develop abnormal brain activity similar to what has been previously found in humans (Fig. 1; Extended Data Fig. 1-2). Quantification of EEG activity through spectral power analysis revealed, much like previous work in AS mouse models (Judson et al., 2016; Born et al., 2017) and in humans with AS (Sidorov et al., 2017), differences in EEG spectral power driven by increased power in δ frequencies. Following early postnatal implantation at P8 or P9, we recorded EEG activity in 1- to 1.5-h recording sessions at ∼1.5 weeks of age. As seen with neonatal rodent recordings from other labs, at this age, cortical EEG activity frequently was discontinuous, which is marked by brief high-amplitude/low-frequency activity mixed with periods of low amplitude activity (Fig. 1*A*). At this point in development, the WT and AS rats showed similar activity with no difference in spectral cortical power (Fig. 1*B*). However, by two weeks of age when cortical EEG activity has matured further and continuous EEG activity is present, there is increased δ power in AS rats because of genotype (*p* < 0.0001) compared with WT littermates with *post hoc* significant differences for multiple frequencies within the δ range (*p* < 0.01 at 1 Hz, *p* < 0.001 at 2–3 Hz, *p* < 0.01 at 4 Hz; Fig. 1*C*,*D*). At three weeks, just after weaning, AS rats maintained an increase in δ cortical power (genotype: *p* < 0.0001; *p* < 0.001 at 1–3 Hz, *p* < 0.05 at 4 Hz; Fig. 1*E*,*F*). The increased δ power in AS rats compared with WT littermates persisted after maturation and into adulthood when measured at the 4.5-month time point (*p* = 0.0036; *p* < 0.05 at 1 Hz; Fig. 1*G*,*H*). Since our approach to analyzing spectral cortical power was using unbiased time of day matching, our initial comparisons were made using data collected during light cycle activity. Representative epochs of cortical EEG activity during the dark cycle, when rats are more likely to be awake than sleeping, was also recorded and analyzed after weaning at three weeks of age (Extended Data Fig. 1-2*A*) and after maturation to adulthood at 4.5 months of age (Extended Data Fig. 1-2*C*). Quantification with spectral analysis showed that the increased δ power in AS compared with WT rats was present during dark cycle activity in juveniles because of genotype (*p* = 0.020; *p* < 0.01 at 1 Hz, *p* < 0.001 at 2 Hz; Extended Data Fig. 1-2*C*) and in adults (*p* < 0.05 at 1 Hz; Extended Data Fig. 1-2*D*).

**Figure 2.**
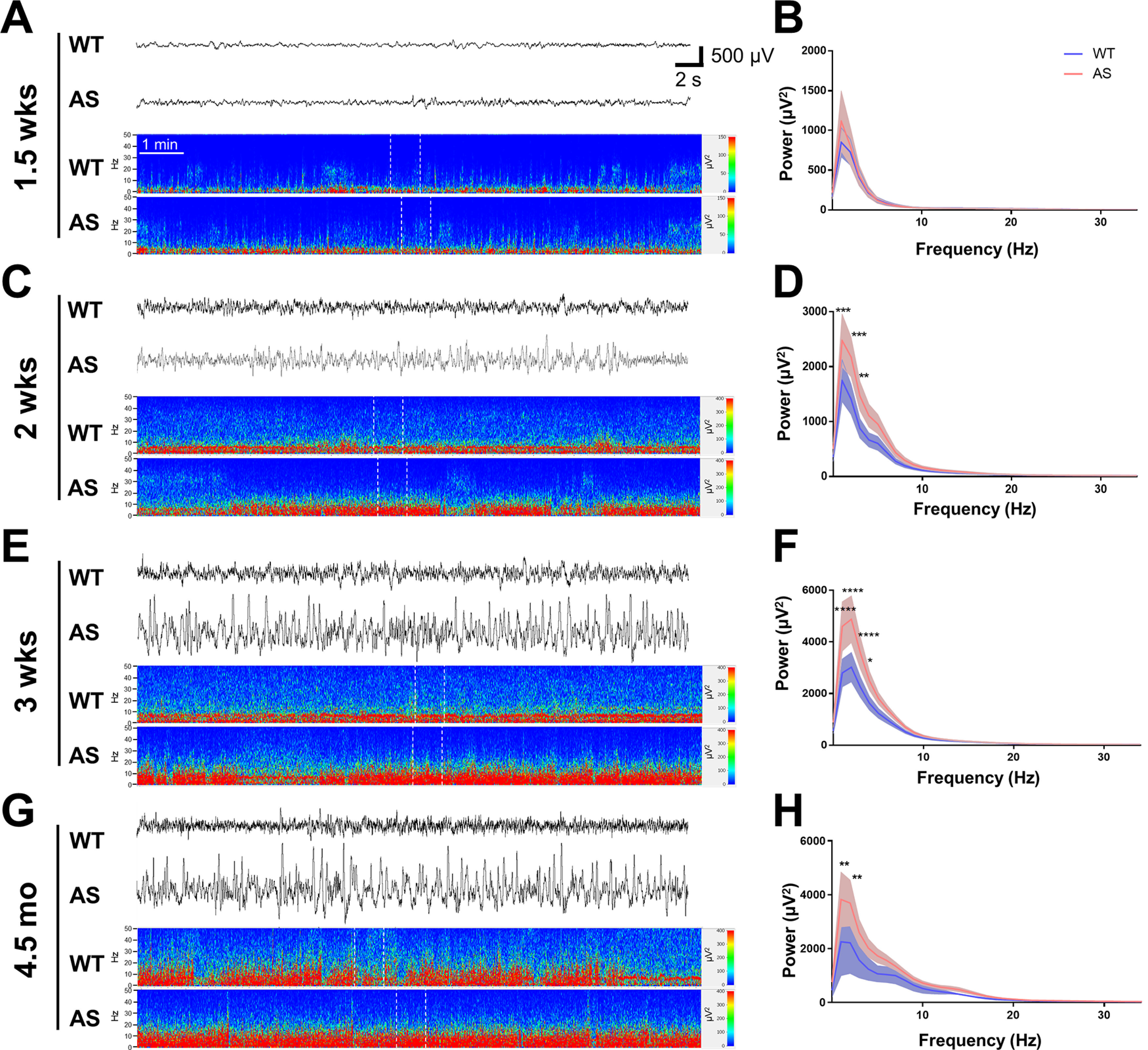
Characterization of hippocampal EEG activity during the light cycle from early development into adulthood. ***A***, ***C***, ***E***, ***G***, Hippocampal EEG traces and power spectra show representative activity from neonates, juveniles, and adult WT and AS rats (with the corresponding EEG activity segment indicated by white dotted lines in each spectrogram), while ***B***, ***D***, ***F***, ***H*** show the age-matched quantification of activity from representative epochs during light cycle activity. ***A***, ***B***, Representative EEG activity and spectral power analysis from 1.5-week-old WT and AS rats show discontinuous activity and spectral power analysis from epochs of continuous activity shows no significant difference in power spectra in the WT and AS rats (*n* = 9). ***C***, ***D***, Representative EEG activity and spectral power analysis at two weeks of age show increased δ power in AS compared with WT rats (*n* = 7–10; *p* < 0.001 at 1–2 Hz, *p* < 0.01 at 3 Hz). ***E***, ***F***, Representative EEG activity and spectral power analysis at three weeks of age show an increase in δ power in AS compared with WT rats (*n* = 7–12; *p* < 0.001 at 1–3 Hz, *p* < 0.05 at 4 Hz). ***G***, ***H***, Representative EEG activity and spectral power analysis show the persistence of increased δ power in adult AS compared with WT rats (*n* = 3–6; *p* < 0.01 at 1–2 Hz); *p < 0.05, **p < 0.01, ***p < 0.001, ****p < 0.0001.

10.1523/ENEURO.0345-20.2020.f1-2 Extended Data Figure 1-2 Cortical EEG activity in juvenile and adult AS and WT rats during the dark cycle. ***A***, ***C***, Cortical EEG traces and power spectra show representative activity from juvenile and adult WT and AS rats (with the corresponding EEG activity segment indicated by white dotted lines in each spectrogram), while ***B***, ***D*** show the age-matched quantification of EEG using power analysis. ***A***, ***B***, Representative EEG activity and spectral power analysis at three weeks of age show an increase in δ power in AS compared to WT rats (*n* = 7–12; *p* < 0.01 at 1 Hz, *p* < 0.001 at 2 Hz). ***C***, ***D***, Representative EEG activity and spectral power analysis at 4.5 months of age show an increase in δ power in AS compared to WT adult rats (*n* = 3–6; *p* < 0.05 at 1 Hz); *p < 0.05, **p < 0.01, ***p < 0.001. Download Figure 1-2, TIF file.

Similar to cortical activity, the early neonatal age at which AS rats were evaluated showed no differences in hippocampal δ power, while qualitative differences could be observed with visual inspection at two weeks of age that persisted into adulthood (Fig. 2; Extended Data Fig. 2-1). At 1.5 weeks of age, discontinuous activity typical of the developing rodent was present with no difference between WT and AS rats with regards to representative hippocampal activity or spectral power analysis (Fig. 2*A*,*B*). Increased δ hippocampal activity was apparent at two weeks of age in AS rats compared with WT littermates because of genotype (*p* = 0.0001) with *post hoc* differences for multiple δ frequencies (*p* < 0.001 at 1–2 Hz, *p* < 0.01 at 3 Hz; Fig. 2*C*,*D*). At three weeks of age, AS rats maintained an increase in hippocampal δ power compared with WT rats (genotype: *p* < 0.0001; *p* < 0.001 at 1–3 Hz, *p* < 0.05 at 4 Hz; Fig. 2*E*,*F*). This pattern of increased δ activity during light cycle hours continued after maturation when measured at 4.5 months of age (genotype: *p* = 0.0016; *p* < 0.01 at 1–2 Hz; Fig. 2*G*,*H*). When we evaluated hippocampal activity recorded during the dark cycle in juvenile and adult rats, we found increased δ power was present at both ages (Extended Data Fig. 2-1*A*,*B* for three-week time point; genotype: *p* < 0.0001; *p* < 0.001 at 1–4 Hz, *p* < 0.05 at 5 Hz; Extended Data Fig. 2-1*C*,*D* for 4.5-month time point; genotype: *p* = 0.0029; *p* < 0.001 at 1 Hz).

10.1523/ENEURO.0345-20.2020.f2-1 Extended Data Figure 2-1 Hippocampal EEG activity from juvenile and adult AS and WT rats during the dark cycle. ***A***, ***C***, Hippocampal EEG traces and power spectra show representative activity from juvenile and adult WT and AS rats (with the corresponding EEG activity segment indicated by white dotted lines in each spectrogram), while ***B***, ***D*** show the age-matched quantification of activity from representative epochs during dark cycle activity. ***A***, ***B***, Representative hippocampal EEG activity and spectral power analysis at three weeks of age highlight an increase in δ power in AS compared to WT (*n* = 7–12; *p* < 0.001 at 1–4 Hz, *p* < 0.05 at 5 Hz). ***C***, ***D***, Representative EEG activity and quantification of spectral power show the persistence of increased δ power in adult AS compared to WT rats (*n* = 3–6; *p* < 0.001 at 1 Hz); *p < 0.05, ***p < 0.001. Download Figure 2-1, TIF file.

### Increased epileptiform activity in AS rats

**Figure 3.**
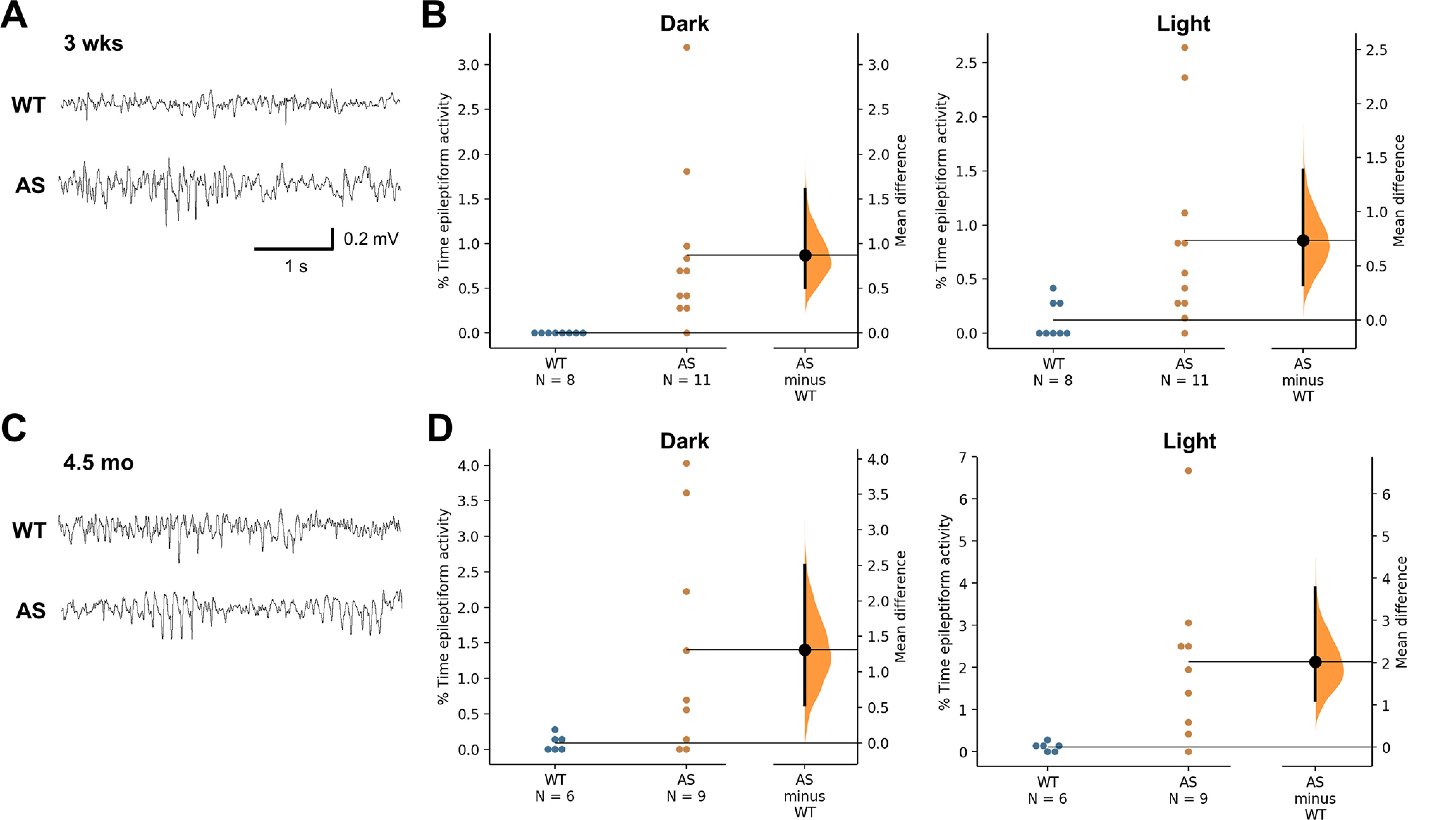
Characterization of epileptiform activity in juvenile and adult AS rats. ***A***, Examples of cortical EEG activity from juvenile WT and AS rats. ***B***, Percentage time spent in epileptiform activity is increased in AS rats at three weeks of age compared with WT rats (*n* = 7–11). ***C***, Examples of cortical EEG activity from adult WT and AS rats. ***D***, Increased percent time in epileptiform activity was found in adult AS compared with WT rats during dark cycle hours (*n* = 6–9).

Previous publications have shown that spontaneous epileptiform activity is present in AS mice, a phenotype that was most pronounced when on the C57BL/6J background (Born et al., 2017). We were interested in whether the AS rats are similarly affected or develop a more pronounced phenotype. Abnormal epileptiform activity was identified in juvenile AS rats (Fig. 3*A*,*B*), including polyspike events of ∼1 s. The percent time during representative epochs of cortical activity that included spike and polyspike events was more frequent in the AS rats at three weeks of age with a mean of 0.86 ± 0.26% time in epileptiform activity compared with limited spikes observed during a comparable amount of time in WT rats during the light cycle epochs (0.12 ± 0.06% time; *p* = 0.020). A similar pattern was found during dark cycle epochs with a mean of 0.87 ± 0.27% time compared with no activity in WT rats (*p* = 0.0097). The percent time that included epileptiform activity was higher in adults evaluated at 4.5 months of age (Fig. 3*C*,*D*), with a mean of 2.13 ± 0.66% time for AS rats compared with 0.11 ± 0.14% time in WT rats during the light cycle epochs (*p* = 0.016). The percent time was significantly increased during dark cycle epochs with a mean of 1.4 ± 0.52% time compared with 0.09 ± 0.05% time in WT rats (*p* = 0.035).

### AS rats show age-dependent increased susceptibility to convulsant stimulation

Since audiogenic stimuli (130-dB alarm) induce seizures in AS mice on a general 129 genetic background, we were interested in whether this phenotype occurred in the AS rat model. Unlike AS mice, the AS rats did not develop generalized AGS at the pup, juvenile, or adult ages tested. We exposed age-matched WT and AS rats to the audiogenic stimulus at two weeks, one month, or 4.5 months of age to test whether there was an age-dependent susceptibility for AGS-induced generalized seizures. Animals did show behaviors that may reflect lower stage seizure activity like freezing and repetitive grooming (Mandel-Brehm et al., 2015). While no differences were seen at two weeks or in the older 4.5-month-age group (Extended Data Fig. 4-1*A*,*C*), at one month of age, AS rats showed a decreased latency to immobile behavior after the start of the alarm noise (*p* = 0.05; Extended Data Fig. 4-1*B*) and an increased time to recover to the first paw movement or grooming after the alarm ended (*p* = 0.0058).

**Figure 4.**
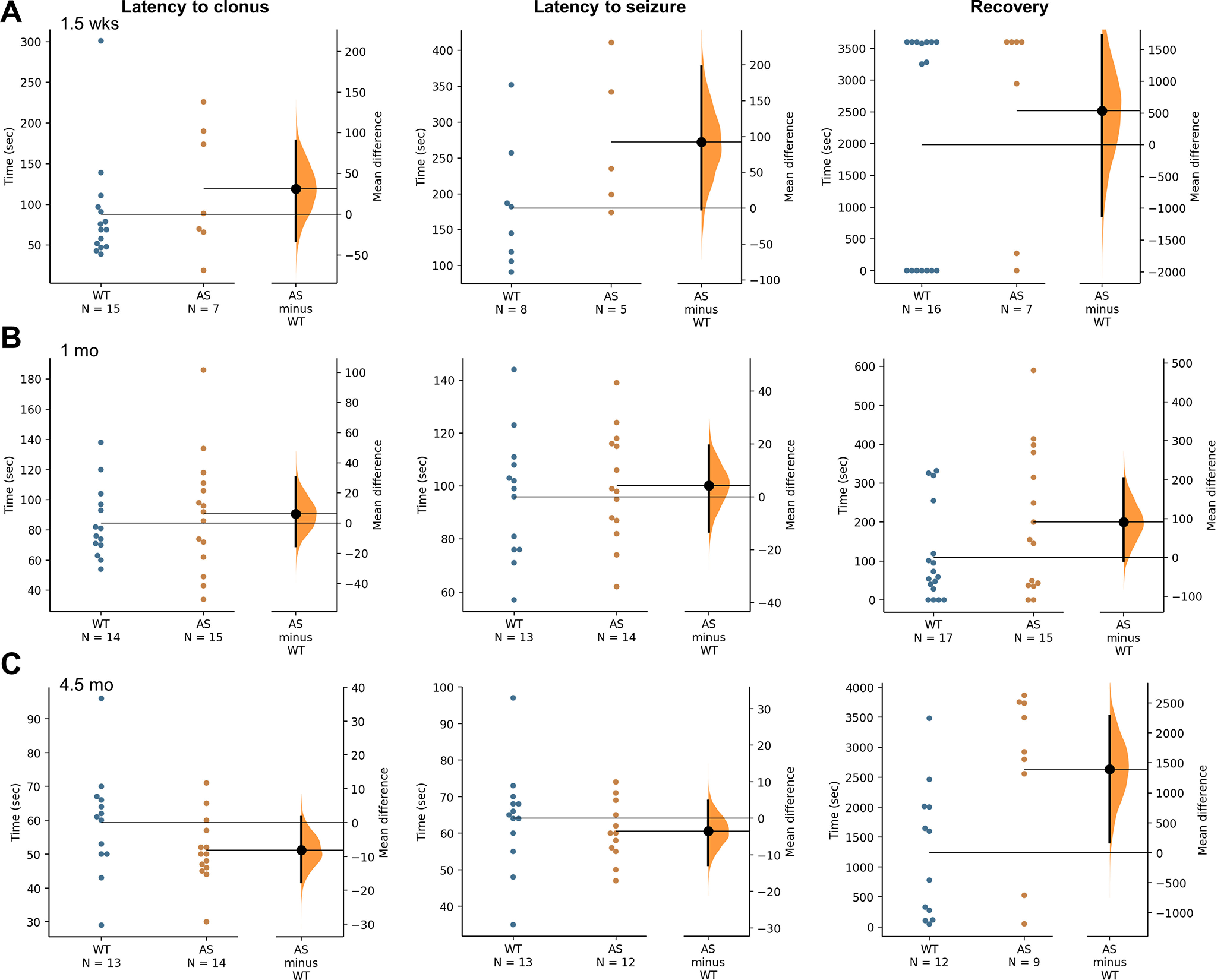
Characterization of susceptibility to PTZ-induced behavioral seizures from early postnatal development into adulthood. ***A***, Latency to first clonus and generalized motor seizure induced by systemic administration of PTZ and measured by behavioral scoring are similar for WT and AS rats at 1.5 weeks of age (*n* = 7–16). ***B***, Juvenile WT and AS rats show a similar latency to first clonus and first generalized motor seizure. The recovery to movement for AS rats is also not significantly different compared with WT littermates (*n* = 17). ***C***, As adults, at 4.5 months of age, the AS rats show similar latencies to first clonus and first seizure compared with WT littermates, however the time to recovery is significantly increased in AS rats at this age (*p* < 0.05, n =14).

10.1523/ENEURO.0345-20.2020.f4-1 Extended Data Figure 4-1 Characterization of behavioral response during audiogenic stimulus. None of the WT or AS rats tested at two weeks (*n* = 6–10), one month (*n* = 12), or 4.5 months (*n* = 14) developed a generalized motor seizure in response to a loud (130 dB) alarm stimulus. ***A***, WT and AS rats exposed to the audiogenic stimuli at two weeks of age showed similar latencies to onset of immobile behavior during the alarm sound and the onset of movement following the end of the alarm sound. ***B***, When tested at one month of age, AS rats showed a decreased latency to immobility while the alarm sounded and a longer time to recover to the first movement following the stop of the alarm. ***C***, At 4.5 months of age, the AS rats no longer showed a difference compared to WT rats in their behavioral response to the audiogenic alarm. Download Figure 4-1, TIF file.

Exposure to systemic administration of the chemoconvulsant PTZ also indicated an age-dependent vulnerability to recover from seizure induction. Following PTZ administration, WT and AS rats were observed for at least 1 h for behavioral seizures and recovery to first movement/grooming following generalized motor seizure behavior. Behavioral scoring did not highlight any change in seizure threshold to PTZ at 1.5 weeks age (Fig. 4*A*) or one month of age (Fig. 4*B*). The percent of WT rats that developed generalized motor seizures at 1.5 weeks age was 56.25% (9/16), compared with 71.4% (5/7) of AS rats. When evaluated at one month of age, 76.5% (13/17) of WT rats progressed to generalized motor seizures, while 82.4% (14/17) of AS rats progressed to this severity. At 4.5 months of age, nearly all animals developed generalized seizures (WT: 100%; 14/14; AS: 92.6%, 13/14) with no difference in the latency to clonus or seizure (Fig. 4*C*). However, the latency to recovery to first paw movement or grooming was significantly increased in AS rats at this age with a latency of 2633 ± 470.4 s compared with a mean of 1238 ± 324.8 s in WT rats (*p* = 0.021; Fig. 4*C*). When comparing the number of animals from AS and WT groups that failed to recover within the 1 h observation period, either as a result of continued seizure behavior or mortality, only 14.3% (2/14) WT rats failed to recover, while 50% (7/14) AS rats failed to recover.

**Figure 5.**
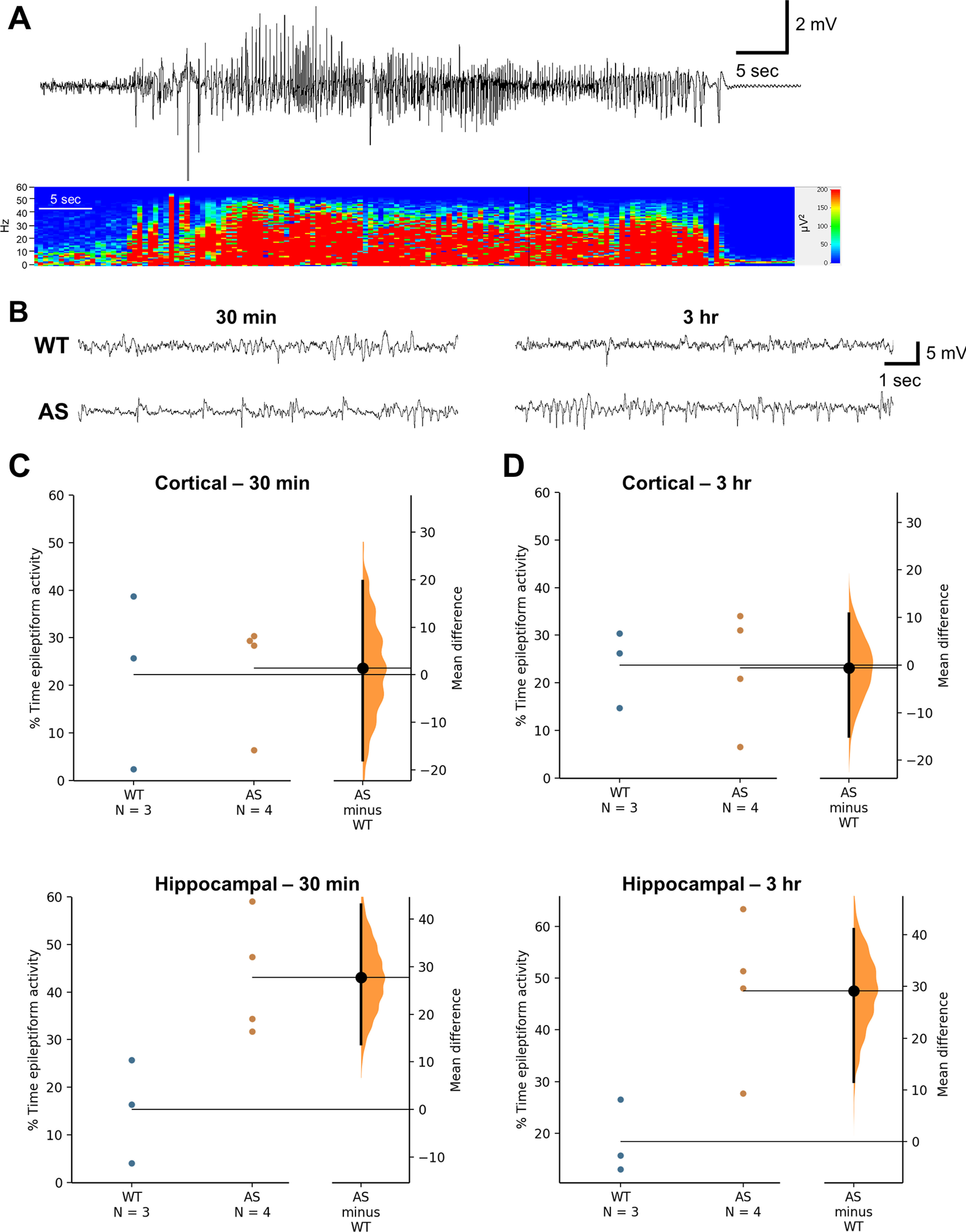
Increased epileptiform activity following a PTZ-induced seizure in adult AS compared with WT rats. ***A***, Representative hippocampal EEG activity and power spectrum of a PTZ-induced single seizure event. ***B***, Representative hippocampal EEG activity in WT and AS rats at 30 min and 3 h following seizure induction. ***C***, AS rats show an increased percent time spent in hippocampal epileptiform activity at 30 min and (***D***) 3 h post-PTZ (*n* = 3–4; *p* < 0.05).

A separate cohort of animals were implanted with cortical and hippocampal electrodes as juveniles and used to evaluate the effect of PTZ at 4.5 months of age by recording EEG activity during seizure induction and the postictal recovery period (Fig. 5*A*,*B*). At 30 min and 3 h post-PTZ, the percent time in epileptiform activity seen in the hippocampus was higher in AS rats compared with WT rats (30 min: *p* = 0.029; 3 h: *p* = 0.027; Fig. 5*C*), while activity evaluated at 1 d post-PTZ was recovered to normal (data not shown). The postictal epileptiform activity may contribute to the lengthier recovery to more normal motor activity observed through behavioral scoring following PTZ-induced seizures in AS rats compared with WT rats. The motor activity measured during behavioral scoring post-PTZ is latency to the first paw movement or grooming episode to provide an objective measurement, however the full recovery to normal motor behavior occurs more gradually over time, and the increased frequency of epileptiform activity seen post-PTZ in AS rats has the potential to disrupt hippocampal rhythms and normal behavioral repertoire.

### Exposure to hyperthermia highlights decreased seizure threshold in AS rats

**Figure 6.**
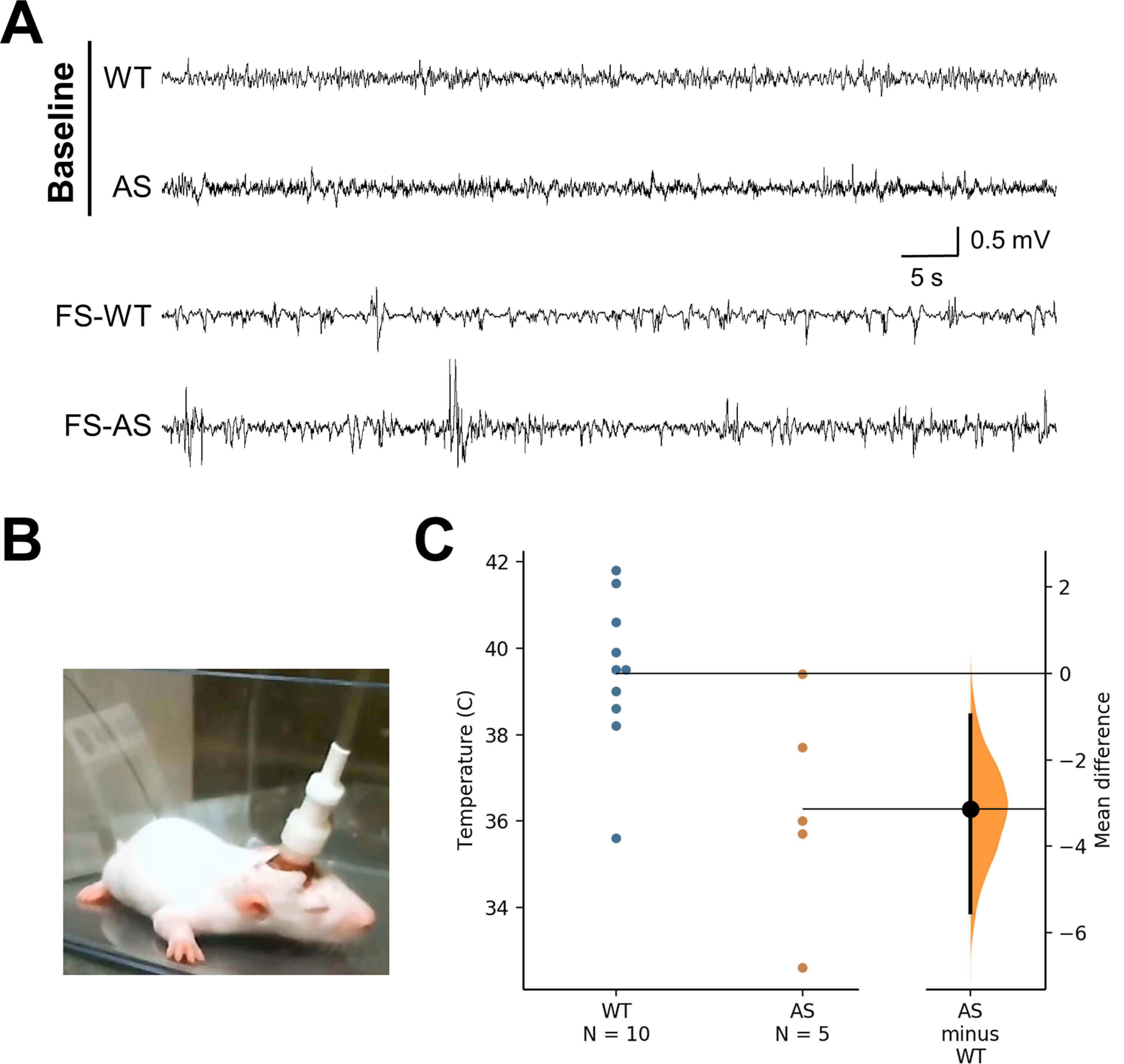
AS rats demonstrate an increased susceptibility to hyperthermia-induced seizures. ***A***, Representative examples of hippocampal EEG at P10–P11 during baseline and hyperthermia induction (FS). Hyperthermia-induced seizures are characterized by high-amplitude, high-frequency burst events (lower two traces: FS-WT, FS-AS). ***B***, Example of EEG-implanted pup prepared for simultaneously evaluation of EEG activity, temperature, and behavior. ***C***, Mean temperature for behavioral onset of hyperthermia-induced seizures is lower for AS compared with WT rats tested at P10–P11 (*n* = 5–10; *p* < 0.05).

In addition to evaluating δ power and epileptiform activity, both of which are strongly associated with AS in humans, we wanted to evaluate seizure threshold using a method with increased clinical relevance to complement our earlier audiogenic and PTZ seizure threshold studies. The use of hyperthermia to induce a controlled FS event is a well-established model and early postnatal FS consistently induces behavioral and electrographic seizures in P9–P11 WT Sprague Dawley rats (Baram et al., 1997; Dutton et al., 2017). We first confirmed that exposure to hyperthermia caused behavioral and electrographic seizures in WT and AS pups (Fig. 6*A*,*B*). Unlike our AGS and PTZ studies, we found that early postnatal AS rats showed an increased susceptibility to hyperthermia as indicated by a lower temperature threshold (36.28 ± 1.13°C) to trigger behavioral seizures compared with WT littermates, which showed the first signs of motor seizures at a mean temperature of 39.42 ± 0.56°C (*p* = 0.015; Fig. 6*C*). There were no significant differences observed in the severity of seizure as measured by maximum Racine scale stage observed (WT: 2.90 ± 0.10 compared with AS: 3.60 ± 0.60) with the most common behavior noted being bilateral forelimb clonus. The duration of time spent in forelimb clonus was not significantly different across genotypes as well (WT: 26.90 ± 8.78 s compared with AS: 53.00 ± 30.77 s).

## Discussion

The development and use of a CRISPR/Cas9 designed genetic rat model for AS is highly advantageous for studies that complement and extend on existing work with AS mouse models, including experiments during an early developmental window, expanded opportunities for behavioral assessments, and susceptibility to a highly-translational method of evoking seizures. The results we report here are important for moving forward future therapeutic studies and can be used for evaluating AS-relevant phenotypic rescue. We have identified novel phenotypes in the AS rat model that are in line with the human disorder, including increased EEG δ power, epileptiform activity, and altered seizure threshold for FSs.

We evaluated cortical and hippocampal EEG activity at multiple ages, ranging from early postnatal at 1.5 weeks to adults at 4.5 months of age, and found increased δ power was present by two weeks of age when the EEG has a more mature continuous pattern and persists at all later ages analyzed. While the time of day affects the pattern of spectral power, likely because of the percent time spent in sleep versus wakefulness at these times in the day, the differences in δ power were seen during both light and dark cycle. Our findings support the use of δ power as a robust, quantitative measure of abnormal brain activity that is more consistently present than ictal events, which can be highly variable, and is present across all species evaluated at this point in time (mouse, rat, and human). Increased δ power has been reported out of multiple labs using different AS mouse models [e.g., *Ube3a ^m−/p+^*, *Ube3aSTOP/p*+ (suppressed neuronal *Ube3a*) and *Ube3aFLOX/p*+*::Gad2-Cre* (GABAergic specific deletion)] on different genetic backgrounds (e.g., C57BL/6J, 129, 129 S2/SvPASCrl; Judson et al., 2016; Born et al., 2017; Sidorov et al., 2017). With studies in humans with AS, abnormal EEG activity has been identified at an early age, and increased cortical δ power has been observed over the past few decades through qualitative visual inspection in a number of reports (Boyd et al., 1988; Valente et al., 2003; Laan and Vein, 2005). In the past few years, quantitative evaluation of EEG activity has highlighted the potential utility for δ power as a quantifiable biomarker for people with AS including unpublished data from our own lab as well as a recent study using data from the AS Natural History Study (NCT00296764) that found abnormal δ power in both deletion and nondeletion genotypes associated with AS (Sidorov et al., 2017; Frohlich et al., 2019a). A similar strategy to identify quantitative electrophysiological biomarkers is also being pursued in the Dup15q syndrome field. Duplications of 15q11.2-q13.1, a region that includes *UBE3A* and three GABA_A_ receptor genes and is highly linked to intellectual disability, autism spectrum disorder, delayed development, and epilepsy, are associated with increased β power, which may be the result of GABAergic pathology in Dup15q syndrome (DiStefano et al., 2016; Frohlich et al., 2016, 2019b). Previous work dissecting the effect of strain background on AS-relevant phenotypes has identified more severe EEG abnormalities in the same genetic background that overall showed the most severe behavioral impairments, and the increased complexities of behavioral evaluations that can be used with rat studies could help facilitate exploration of whether neurobehavioral impairments correlate with abnormal spectral power.

Epilepsy frequently occurs in genetic disorders as part of a larger cluster of symptoms, such as abnormal interictal brain activity, disordered sleep, mood disorders, and cognitive dysfunction. In the present study, while epileptiform activity was found in juvenile AS rats and at a higher percent of time in the adult group, spontaneous seizures were not noted. However, observations from the AS rat colony suggest that a spontaneous seizure phenotype may develop at an older age (data not shown). Absence-like events and generalized motor seizures have been observed in older AS animals in their home cages, including an AS animal that developed severe generalized motor seizures and experienced early mortality, which is an area of future investigation. In line with the increased susceptibility to AGS that has been found in existing AS mouse models, we tested whether AS rats were also vulnerable to this type of evoked seizure. In contrast with the high percentage of AS mice on the 129 background that develop generalized motor seizures (Jiang et al., 1998b; Mandel-Brehm et al., 2015; Born et al., 2017), we did not observe motor seizures in AS rats tested at different ages or in the WT rats tested, although we found a transient altered audiogenic-induced freezing behavior in AS rats at one month of age that may represent less severe seizure behavior that required an increased recovery to normal behavior. Another consideration is that this test is highly strain dependent in AS mice, and the genetic background of the Sprague Dawley rats may provide protection against convulsant stimulation. The results from our PTZ-induced seizure studies indicate that while a similar percent of adult animals for both WT and AS genotypes will develop generalized seizures, a longer length of time is necessary and an increased frequency of failure to recover to more normal behavior occurs in AS rats compared with WT. Data from our EEG-implanted cohort suggests that there is increased epileptiform activity during this extended recovery, and that there are additive short-term effects of seizure events when combined with the underlying genetic disorder. While activity returns to normal within 24 h, the increased epileptiform activity found in AS rats at 30 min and 3 h after the PTZ-induced seizure has the potential to impact learning and memory behavior. Extending this work to determine whether there is an increased risk in AS for developing long-term changes as a result of an acute seizure event, including threshold to subsequent seizures later on would have translational relevance.

Our finding that the AS rats were susceptible to hyperthermia-evoked behavioral seizures at a lower temperature than WT littermates suggests this model recapitulates a sensitivity to fever that has been noted, but not well-studied, in previous clinical reports. To the best of our knowledge, the early postnatal decreased threshold to hyperthermia-induced seizures reported here in naive AS rats is the first AS model that shows sensitivity to increased body temperature without requiring previous exposure to flurothyl kindling, which has been shown to reduce seizure threshold in AS mice (Gu et al., 2019). AS children are more vulnerable to FSs during illness in infancy compared with the general population (3–5%; Hauser, 1994), may experience FS as the first seizure in the development of epilepsy (20–73%; Viani et al., 1995; Laan et al., 1997), and remain at higher risk for FS over an extended developmental window (Galvan-Manso et al., 2005; Valente et al., 2006). While there is an established link between seizures and inflammation (Vezzani et al., 2011; de Vries et al., 2016), it is unknown whether inflammation is increased in many developmental disorders, such as AS, or if insults like illness or seizures trigger an exaggerated response. Although many FS are thought to be benign (Ellenberg and Nelson, 1978; Verity et al., 1998; Shinnar, 1998), severe FS can result in status epilepticus that is difficult to stabilize and requires steroids for recovery. Understanding this relationship may help guide improved therapeutic strategies during infection and fever and episodes of status epilepticus in the AS population. Moreover, given the current momentum of ongoing and future clinical trials for therapeutic approaches that will be delivered directly to the central nervous system like antisense oligonucleotides (ASOs) or genetic therapies, which can trigger inflammation, the response to febrile conditions will be important to understand.

The rat as a model animal provides a number of new opportunities and advantages compared with existing mouse models, including body and brain size, which has facilitated early developmental studies, such as our EEG recordings, more complex behavioral testing, and potentially future longitudinal studies examining quantitative biomarkers in blood or CSF, which can be more easily done through non-terminal collections with rats compared with mice. The use of rats as a model animal provides the opportunity to mimic the human disorder more closely, and the use of an outbred genetic background like Sprague Dawley allows for more genetic variability, similar to the human population. In addition to the results reported here, a number of behavioral deficits related to developmental delays, motor deficits and abnormal gait, abnormal social behavior, and impaired learning and memory have been identified in this model that are similar to those found in humans with AS. This specific rat model of AS includes a complete deletion of the maternal allele of *Ube3a*, which is similar to the disruption of *UBE3A* found in many people with AS and will be beneficial for testing current and future treatment strategies for AS, many of which are targeting functional replacement or activation of UBE3A or *UBE3A*, respectively in the central nervous system. While this study focuses on the loss of maternal *Ube3a*, future studies modeling in rats the larger maternal deletion that encompasses other genes in the 15q11-q13 region frequently found in AS patients have the potential to elicit a more severe phenotype, similar to what has been found in humans and mice (Jiang et al., 2010; Frohlich et al., 2019a). Previous studies from other labs using AS mouse models have shown the promise of a variety of different targeted approaches, including ASOs, adeno-associated viral vectors, and artificial transcription factors among other innovative strategies to achieve functional correction of UBE3A expression in the brain (Daily et al., 2011; Meng et al., 2013; Bailus et al., 2016). Therapeutic interventions to reduce the severity of phenotypes through dietary approaches or pharmacological means are also highly active areas of development for the treatment of AS that can benefit from using the AS rat model for preclinical studies (Ciarlone et al., 2016; Grocott et al., 2017).

There is an urgent need for developing new and more effective treatment options to improve or rescue aspects of the AS phenotype. The maternal *Ube3a* deletion AS rat model that we used for the studies reported here and that were initially reported in recent publications (Berg et al., 2020; Dodge et al., 2020), displays a number of AS-relevant phenotypes that are of great interest for understanding and targeting for therapeutic interventions. We have identified new phenotypes in the AS rat model through our EEG activity and seizure threshold studies. Our results are highly relevant to the human disorder and demonstrate an increase in cortical and hippocampal δ power, epileptiform activity, and altered response to evoked seizures. Our work adds support for the utility of the AS rat model and is informative for future studies to identify new biomarkers and develop and evaluate new therapeutic strategies.

## Synthesis

Reviewing Editor: Deanna Smith, University of South Carolina Decisions are customarily a result of the Reviewing Editor and the peer reviewers coming together and discussing their recommendations until a consensus is reached. When revisions are invited, a fact-based synthesis statement explaining their decision and outlining what is needed to prepare a revision will be listed below. The following reviewer(s) agreed to reveal their identity: Ben Philpot, Eric Levine.

Both authors agree that the data presented are of value for the Angelman Syndrome field, and in particular will clarify the seizure phenotypes associated with rodent AS models. The availability of a rat model to complement the mouse model is seen as important and should help future preclinical trials in AS. The reviewers point out some issues that could strengthen the manuscript. These are included below:

Reviewer 1:

This manuscript characterized the EEG and seizure related outcomes across development in a rat model of Angelman syndrome. The similarities of EEG abnormality and seizure sensitivity shared among rat and human make it a powerful preclinical rodent model for AS study. A major contribution to the field is the study of EEG phenotypes in early development in AS rodent model, as to date there have only been cursory characterizations. This is a strength of the manuscript, as this information is needed to improve preclinical therapeutic studies. There are some concerns that need to be addressed, most or all of which are easily addressable:

1) The "epileptiform activity" is an extremely vague concept in this manuscript, and it needs to be clearly defined. Based on the information provided in the methods, it includes "spikes" and "polyspikes", however, these definitions do not reflect clinical epileptiform discharges, which include 3-Hz spike-and-wave, slow spike-and-wave, atypical paroxysmal fast activity, and hypsarrhythmic patterns.

2) How do the rats behave during "epileptiform activity"? Normal behaviors like grooming and eating typically generate "spikes" and "polyspikes", which could be classified by the authors as "epileptiform activity". Please clarify.

3) The representative traces showed in Figure 3 A and C do not appear to represent typical epileptiform activity

4) The definition of epileptiform activity is important, particularly in light of the fact that the WT rats also spent time in "epileptiform activity". Is this expected, or does this suggest that there are some detection errors?

5) The introduction or discussion could benefit from a description of the types of epileptiform activity that is observed in children and adults with AS.

Minor concerns:

1. Abstract, line 9-11, please rephrase "While EEG power was similar to wildtype (WT) at 1.5 weeks postnatally at all other ages analyzed, our findings were similar to AS mice and humans with AS with significantly increased delta power." Punctuation seems missing.

2. Page 3, line 57-60. The author wrote "Translating pre-clinical studies to therapeutics has been made more difficult by the limitations of mouse models, as Ube3a maternally deficient mice show strain-dependent, inconsistent, and varying seizure and behavioral phenotypes (Jiang et al., 1998b; Huang et al., 2013b; Mandel-Brehm et al., 2015; Born et al., 2017). Thus, highlighting a need for pre-clinical models that reliably mimic features of AS." Here, the author should highlight the outbred nature of the SD rats used in this study. Also, it has yet to be determined if other rat backgrounds show the same phenotypes, or if other investigators obtain similar results as those presented here.

3. Page 16, line 263 "on different genetic backgrounds (e.g. C57BL/6J, 129, 129 S2/SvPASCrl)". Please specify the substrain of "129"

4. Figure 1 and 2. Please show matched representative EEG trace and spectrogram. And/or please specify the segment in the spectrogram where the EEG trace represents.

5. Figure 1 and 2. Not clear what each of the significance marks (*) represent (1, 2, 3, 4 Hz)? Please specify. Please also quantify EEG powers in each wave bands (beta, theta, alpha...)

6. "The post-ictal epileptiform activity may contribute to the lengthier recovery to more normal motor activity observed through behavioral scoring following PTZ-seizure in AS rats compared to WT rats." Please discuss the presence of "epileptiform activity" at 3 hr post PTZ (Figure 5C, right panel), which is long after the behavioral recovery in AS rat (Figure 4C, right panel).

7. Figure 6A, the thickness of the representative traces differ, making it somewhat difficult to compare.

8. Please quantify hyperthermia-induced seizure severity and duration of WT vs AS rat.

Reviewer 2:

This manuscript characterizes EEG activity, epileptiform discharges, and seizure susceptibility in a novel rat model of Angelman syndrome (AS) that was recently reported in an earlier publication. This is a significant study because existing mouse models of AS have shown mixed results regarding seizure phenotypes depending on the background strain, and the use of a rat model offers several distinct advantages. The studies are well designed and the manuscript is well written. The results present a clear increase in EEG delta power in AS rats, matching previous results from AS mice as well as humans. Seizure susceptibility in the AS rat is minimally affected with PTZ-induced and audiogenic seizures, but a difference in threshold is reported for febrile seizures. These results are an important contribution to our understanding of the role of UBE3A in AS pathophysiology.

An important issue that the authors need to address is with regard to modeling AS with a specific UBE3A deletion vs. a large deletion of the 15q11-q13 region. The authors state multiple times that UBE3A deletion is the most common cause of AS, however 70-80% of Angelman patients have a q11-13 deletion, which encompasses UBE3A and several other non-imprinted genes, including the GABA-A receptor cluster. Only a minority of AS patients have loss of function specific to UBE3A. Individuals with the typical large deletion have a more severe phenotype than patients with mutation/loss of UBE3A alone, especially with regards to seizures, which are less prominent in patients with specific UBE3A disruption. This should be clarified and this issue should be discussed in relation to the phenotype observed in these animals.

Minor points:

- The authors state that "Exposure to systemic administration of the chemoconvulsant pentylenetetrazol (PTZ) also indicated an age-dependent vulnerability to seizure induction", however it is not clear how the change in recovery time indicates a change in seizure induction.

- The authors report that "AS rats showed an increased susceptibility to hyperthermia and a lower temperature threshold". It seems that the lower threshold is the only evidence of increased susceptibility, so a more accurate statement would be "increased susceptibility to hyperthermia as indicated by a lower temperature threshold".

- The Y axis label for Fig 4B recovery time seems to be mislabeled.
