# Supplementary figures and images for: Early Developmental EEG and Seizure Phenotypes in a Full Gene Deletion of Ubiquitin Protein Ligase E3A Rat Model of Angelman Syndrome
Source: eNeuro. 2021 Mar 23;8(2):ENEURO.0345-20.2020. doi: 10.1523/ENEURO.0345-20.2020 (PMC8114899; doi:10.1523/ENEURO.0345-20.2020)

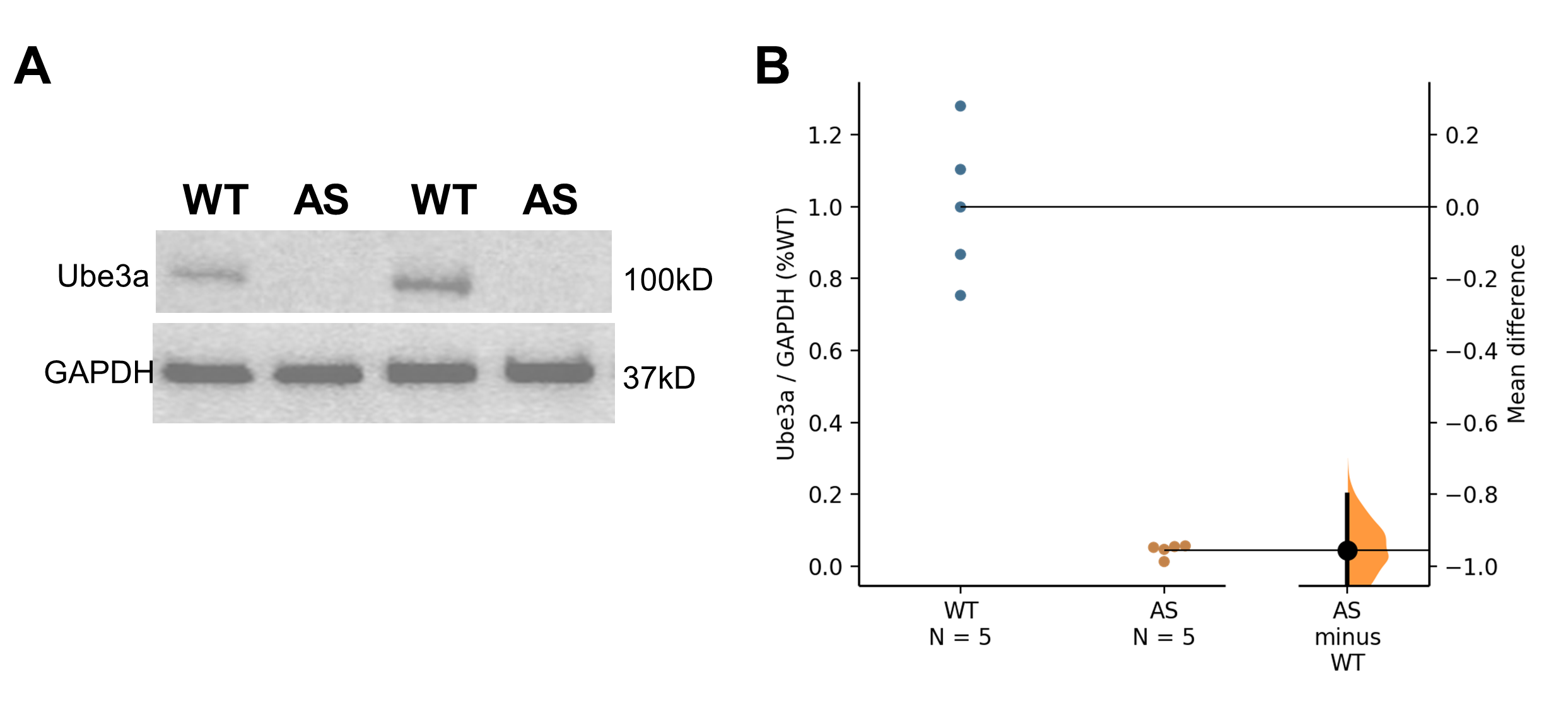

Supplement: Extended Data Figure 1-1 — Confirmation of Ube3a protein expression loss in the Ube3a maternal deficiency AS rat model. A, Western blotting for Ube3a (Sigma E8655; 1:1000) with GAPDH used as a loading control confirms loss of expression in adult AS rat hippocampus. B, The expression level of Ube3a is significantly decreased in hippocampal tissue from adult AS rats compared to age-matched WT littermates (n = 5, p < 0.0001). Download Figure 1-1, TIF file. [file enu-eN-NWR-0345-20-s02.tif]

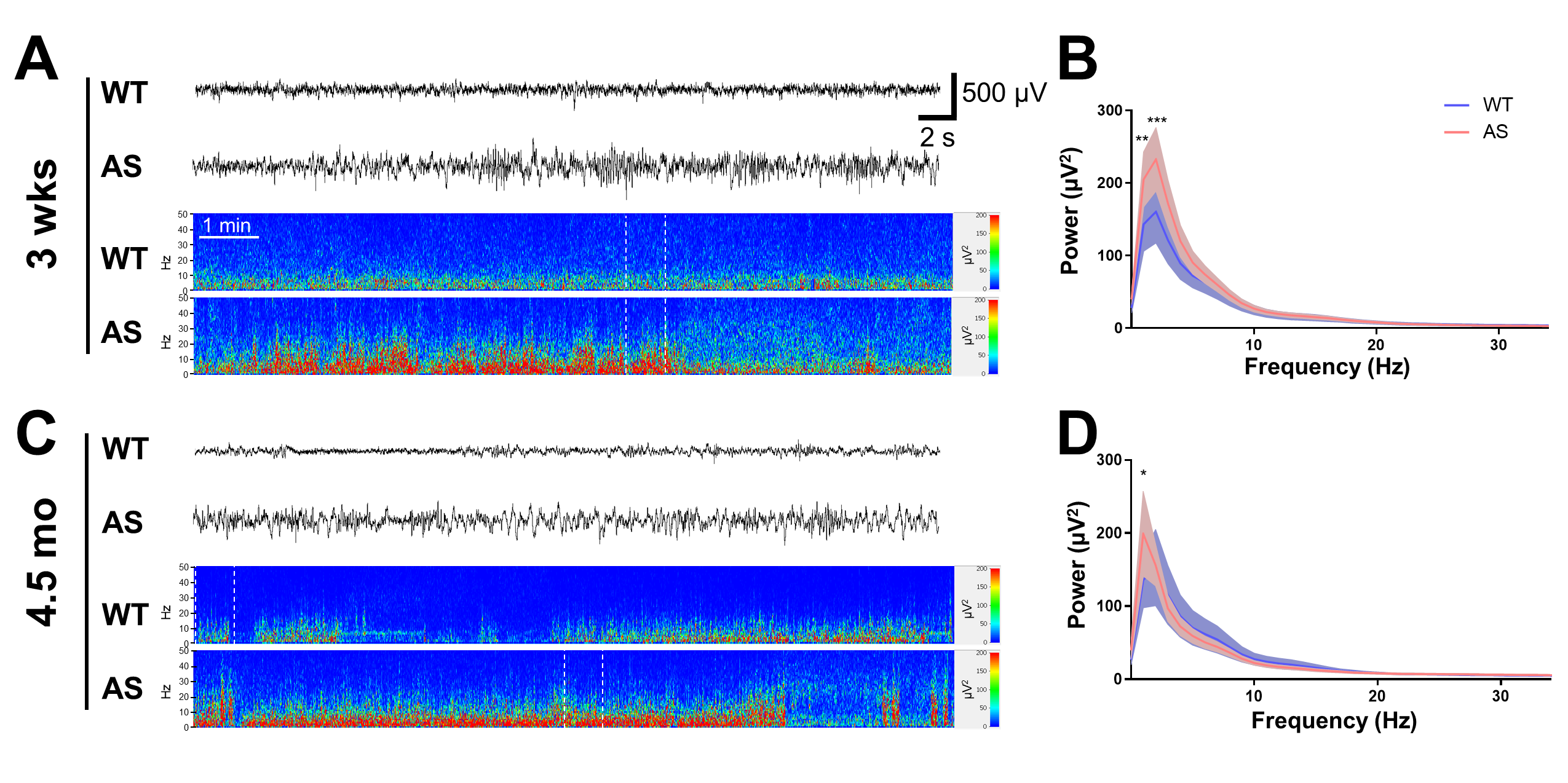

Supplement: Extended Data Figure 1-2 — Cortical EEG activity in juvenile and adult AS and WT rats during the dark cycle. A, C, Cortical EEG traces and power spectra show representative activity from juvenile and adult WT and AS rats (with the corresponding EEG activity segment indicated by white dotted lines in each spectrogram), while B, D show the age-matched quantification of EEG using power analysis. A, B, Representative EEG activity and spectral power analysis at three weeks of age show an increase in δ power in AS compared to WT rats (n = 7–12; p < 0.01 at 1 Hz, p < 0.001 at 2 Hz). C, D, Representative EEG activity and spectral power analysis at 4.5 months of age show an increase in δ power in AS compared to WT adult rats (n = 3–6; p < 0.05 at 1 Hz); *p < 0.05, **p < 0.01, ***p < 0.001. Download Figure 1-2, TIF file. [file enu-eN-NWR-0345-20-s03.tif]

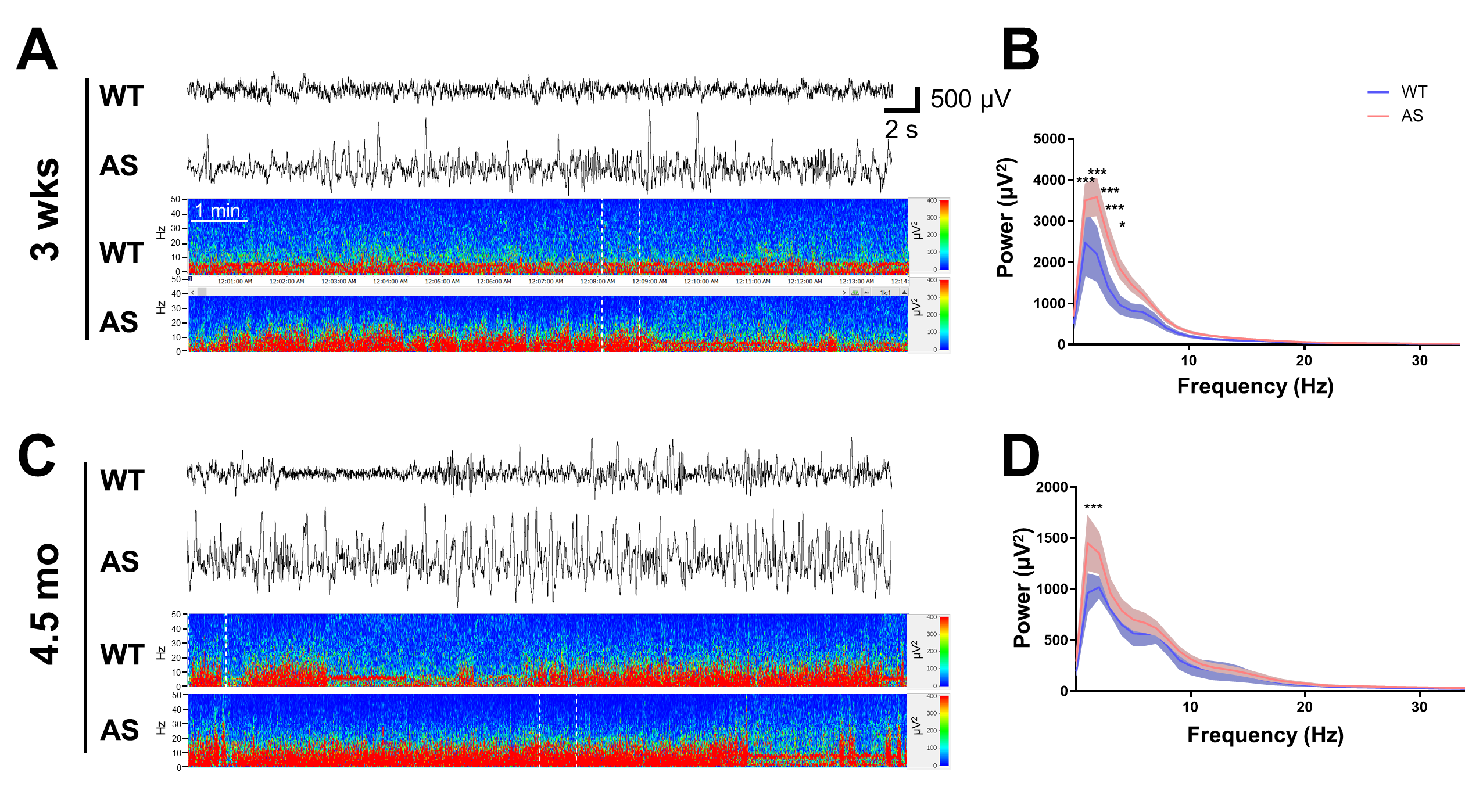

Supplement: Extended Data Figure 2-1 — Hippocampal EEG activity from juvenile and adult AS and WT rats during the dark cycle. A, C, Hippocampal EEG traces and power spectra show representative activity from juvenile and adult WT and AS rats (with the corresponding EEG activity segment indicated by white dotted lines in each spectrogram), while B, D show the age-matched quantification of activity from representative epochs during dark cycle activity. A, B, Representative hippocampal EEG activity and spectral power analysis at three weeks of age highlight an increase in δ power in AS compared to WT (n = 7–12; p < 0.001 at 1–4 Hz, p < 0.05 at 5 Hz). C, D, Representative EEG activity and quantification of spectral power show the persistence of increased δ power in adult AS compared to WT rats (n = 3–6; p < 0.001 at 1 Hz); *p < 0.05, ***p < 0.001. Download Figure 2-1, TIF file. [file enu-eN-NWR-0345-20-s04.tif]

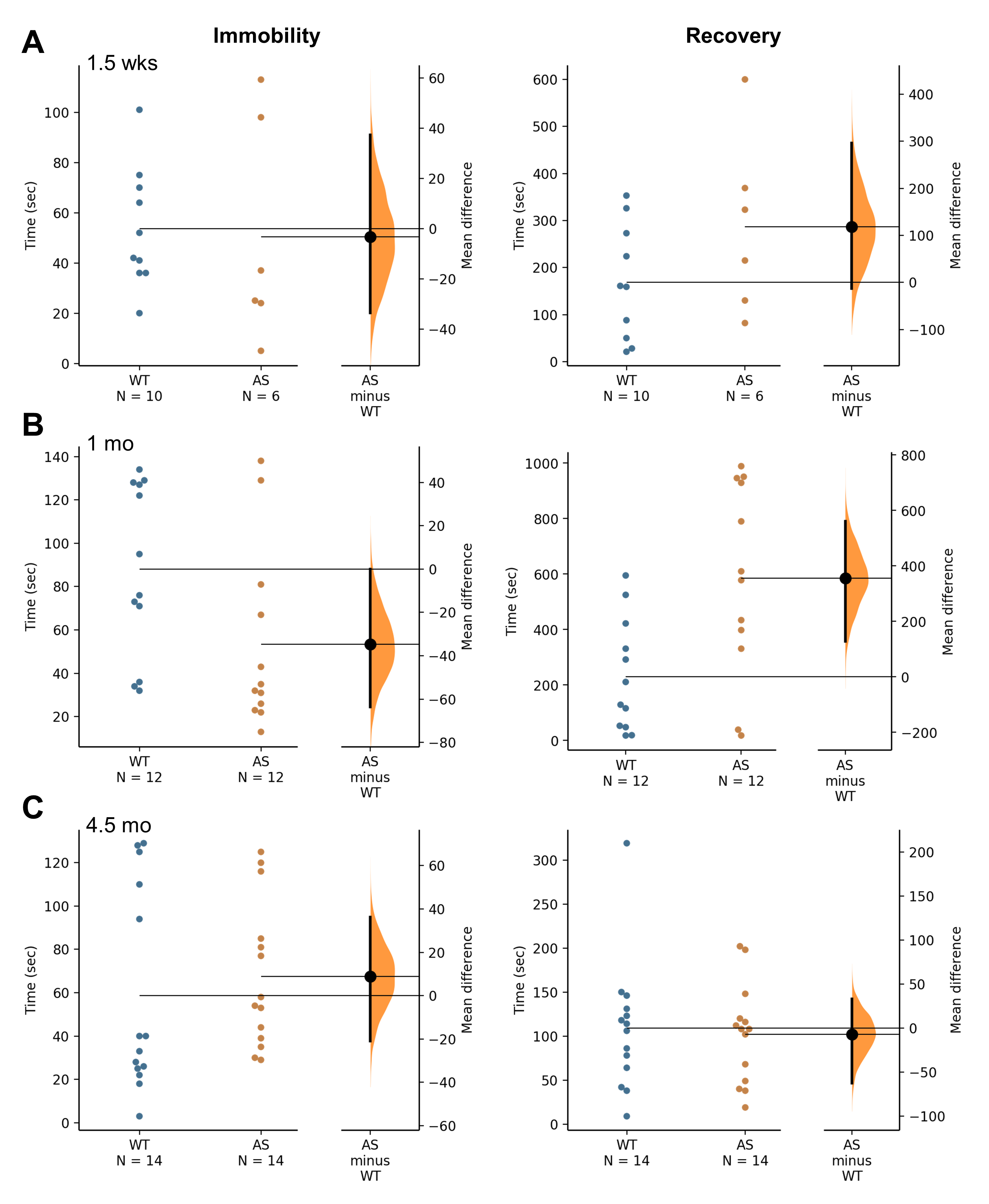

Supplement: Extended Data Figure 4-1 — Characterization of behavioral response during audiogenic stimulus. None of the WT or AS rats tested at two weeks (n = 6–10), one month (n = 12), or 4.5 months (n = 14) developed a generalized motor seizure in response to a loud (130 dB) alarm stimulus. A, WT and AS rats exposed to the audiogenic stimuli at two weeks of age showed similar latencies to onset of immobile behavior during the alarm sound and the onset of movement following the end of the alarm sound. B, When tested at one month of age, AS rats showed a decreased latency to immobility while the alarm sounded and a longer time to recover to the first movement following the stop of the alarm. C, At 4.5 months of age, the AS rats no longer showed a difference compared to WT rats in their behavioral response to the audiogenic alarm. Download Figure 4-1, TIF file. [file enu-eN-NWR-0345-20-s05.tif]
